# Nucleoside‐Modified mRNA Encoding Alpha‐Galactosidase A Ameliorates Fabry Disease Phenotypes in Human IPSC‐Derived Cardiomyocytes

**DOI:** 10.1002/advs.77151

**Published:** 2026-08-13

**Authors:** Malte Juchem, Lea Oehlsen, Sedef Ersoy, Jia Li Ye, Natalie Weber, Elisa Mohr, Wilson Agyapong, Junqing Liu, Maximilian Fuchs, Ke Xiao, Christopher Jahn, Reto Eggenschwiler, Tobias Cantz, Julia Beimdiek, Falk F. R. Buettner, Theresia Kraft, Jan Hegermann, Ivonne J. Knorr, Lisa Ernst, Linda Feldbrügge, Resa Puffert, Kevin Schmidt, Christian Bär, Jeannine Hoepfner, Thomas Thum

**Affiliations:** ^1^ Institute of Molecular and Translational Therapeutic Strategies (IMTTS) Hannover Medical School Hannover Germany; ^2^ Fraunhofer Institute for Toxicology and Experimental Medicine (ITEM) Hannover Germany; ^3^ Center of Translational Regenerative Medicine Hannover Medical School Hannover Germany; ^4^ Dean's Office for Academic Career Development nextGENERATION Medical Scientist Program Hannover Medical School Hannover Germany; ^5^ Department of Gastroenterology Hepatology, Infectious Diseases and Endocrinology Hannover Medical School Hannover Germany; ^6^ Proteomics, Institute of Theoretical Medicine Faculty of Medicine University of Augsburg Augsburg Germany; ^7^ Institute of Molecular and Cell Physiology Hannover Medical School Hannover Germany; ^8^ Institute of Functional and Applied Anatomy Research Core Unit Electron Microscopy Hannover Medical School Hannover Germany; ^9^ Institute For Laboratory Animal Science and Experimental Surgery Faculty of Medicine RWTH Aachen University Aachen Germany; ^10^ Department of General Visceral and Transplant Surgery Hannover Medical School Hannover Germany; ^11^ Fraunhofer Cluster of Excellence Immune‐Mediated Diseases Hannover Germany

**Keywords:** calcium handling, cardiomyopathy, Fabry disease, globotriaosylceramide, lipid based nanoparticles, mRNA therapy, phospholamban hyperphosphorylation, reactive oxygen species

## Abstract

The lysosomal storage disorder Fabry disease results from α‐galactosidase A deficiency, leading to excessive glycosphingolipid substrate accumulation, primarily globotriaosylceramide (Gb3). While the underlying molecular mechanisms remain elusive, multi‐systemic complications ultimately culminate in premature death, with heart failure being the leading cause of death. Current treatment options fail to treat Fabry disease adequately and only delay its progression. Preclinical studies on an alternative approach, systemic delivery of nucleoside‐modified *GLA* mRNA (modGLA), suggest improved effectiveness over existing therapies in reducing glycosphingolipid levels in the heart. It remains unclear whether modGLA can rescue Fabry cardiomyopathy phenotypes at the cellular level, which are not faithfully recapitulated in current animal models. To address this, we investigated characteristic phenotypes in two new models of Fabry cardiomyopathy utilizing human iPSC‐derived cardiomyocytes in transcriptomic and functional analyses. These human Fabry disease cardiomyocytes displayed broad transcriptional dysregulation, apoptosis, mitochondrial dysfunction, impaired reactive oxygen species handling, altered contractility, and enhanced calcium transient decay parameters. Mechanistically, phospholamban hyperphosphorylation may contribute to this calcium dysregulation. Consistently, modGLA therapy restored α‐galactosidase A activity, reduced glycosphingolipid deposition, and normalized molecular alterations, including phospholamban hyperphosphorylation and calcium decay parameters, supporting modGLA as a promising therapeutic strategy for Fabry disease.

## Graphical abstract

Human iPSC‐derived Fabry cardiomyocytes exhibited broad transcriptional dysregulation, apoptosis, mitochondrial dysfunction, impaired reactive oxygen species handling, altered contractility, and abnormal calcium transient decay, potentially mediated by phospholamban hyperphosphorylation. modGLA therapy restored α‐galactosidase A activity, reduced glycosphingolipid accumulation, and normalized molecular and functional abnormalities, supporting its potential as a therapeutic strategy for Fabry cardiomyopathy (Created in BioRender. Hoepfner, J. (2026) https://BioRender.com/dfnb59i).

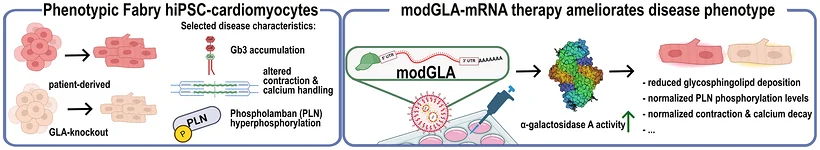

## 1 Introduction

Fabry disease (OMIM #301500) is an X‐linked lysosomal storage disorder that results from deficiency of the lysosomal enzyme α‐galactosidase A, encoded by pathogenic *GLA* variants [1]. Consequently, the impaired metabolism of its glycosphingolipid substrates, primarily globotriaosylceramide (Gb3) [2], causes their build‐up throughout patients’ tissues. This glycosphingolipid accumulation is discussed to modulate secondary biochemical effects including membrane lipid composition, protein trafficking and mitochondrial function [3], which then progressively manifest as a multisystemic disorder, most severely affecting the cerebral, renal, and cardiovascular system. The primary cause of death are cardiovascular complications both in affected men and women [4]. Typically observed cardiac phenotypes include left ventricular hypertrophy, arrhythmias, myocardial fibrosis and eventually heart failure [5]. On cellular and tissue level oxidative stress and apoptosis [6], mitochondrial dysfunction and consequently impaired cardiac energy metabolism as well as inflammation [7], have been reported.

Unfortunately, Fabry disease remains inadequately addressed by the existing treatment options. Although available therapeutic options for Fabry disease have been demonstrated to temporarily stall disease progression, patients continue to experience a considerable disease burden that culminates in premature death [8, 9]. While enzyme replacement therapy (ERT) appears to slow disease progression it fails to remove Gb3 from affected cardiomyocytes or to ameliorate heart dysfunction even after follow‐up of 20 years [10, 11]. The only approved alternative is based on pharmacological chaperones, which are only available to a subset of patients with amenable mutations [12].

The Fabry pathomechanism remains insufficiently understood. Unravelling the underlying molecular mechanisms might allow the development of novel treatment approaches. However, the established animal models including *GLA*‐KO mice [13, 14] and rats [15] fail to mirror the consequences of *GLA* deficiency observed in Fabry patients, particularly the cardiac manifestation. Consequently, alternative models are required to study disease mechanisms and test the effectiveness of novel treatments in a relevant and translational system. To approach this, we aimed to establish such an alternative, a general and patient‐independent in vitro Fabry disease model by knockout of *GLA* in hiPSCs derived from a healthy donor. Subsequently, we utilized this model system to investigate the effectiveness of the promising novel candidate‐treatment ‘nucleoside‐modified *GLA* mRNA (modGLA)’ in ameliorating Fabry‐typical cellular disease phenotypes.

The SARS‐CoV 2 pandemic illustrated the immense potential of RNA‐based drugs. Nucleoside modifications may have partly facilitated their wide‐spread use [16]. In the context of Fabry disease, preclinical studies using *GLA* mRNA have shown an improved therapeutic efficacy in the hearts and kidneys of *GLA*‐KO mice, specifically regarding a reduction of Gb3 and lyso‐Gb3 levels, when compared to ERT alone, ERT and chaperone therapy combined, or other gene therapy approaches [17]. Yet, established animal models fail to recapitulate the complex cellular pathology of Fabry disease. In vitro data for modGLA therapy in human Fabry cardiomyocytes remain limited to reduction of Gb3 deposition and partial normalization of lysosomal protein levels [18]. Here, we investigate whether nucleoside‐modified *GLA* mRNA can rescue disease‐relevant cellular phenotypes beyond substrate accumulation and provide mechanistic insight into cardiomyocyte dysfunction in Fabry disease.

## 2 Results

### 2.1 Loss of *GLA* Results in Accumulation of the α‐Galactosidase A Substrate Globotriaosylceramide in hiPSC‐Derived Cardiomyocytes

To investigate the specific effects of functional *GLA* loss underlying the cardiac Fabry disease manifestation, while minimizing confounding genetic factors putatively present in patient‐derived hiPSC‐based in vitro disease models, we previously generated *GLA*‐knockout (KO) hiPSCs from a healthy donor‐derived *GLA* wild type (WT) parental line using CRISPR/Cas9. Off‐target assessment of the CRISPR‐generated *GLA*‐knockout line was performed during the original characterization of this cell line and is described in detail therein [19, 20]. In the present study, both *GLA*‐KO and parental WT hiPSC lines readily differentiated into hiPSC‐derived cardiomyocytes (CMs) as indicated by 95.36 (0.93)% and 94.63 (0.68)% cardiac troponin T‐positive cells for WT and *GLA*‐KO CMs, respectively (Figure 1B). As expected, *GLA*‐KO CMs were α‐galactosidase A enzyme deficient (Figure 1D) which resulted in progressive accumulation of its glycosphingolipid substrate Gb3 (Figure 1C). Since myofibrillolysis has been anecdotally reported in endomyocardial biopsies from Fabry patients [21], we performed transmission electron microscopy to analyze sarcomere ultrastructure. However, no apparent differences between the models were observed in 65‐day‐old CMs (Figure 1E).

**FIGURE 1:**
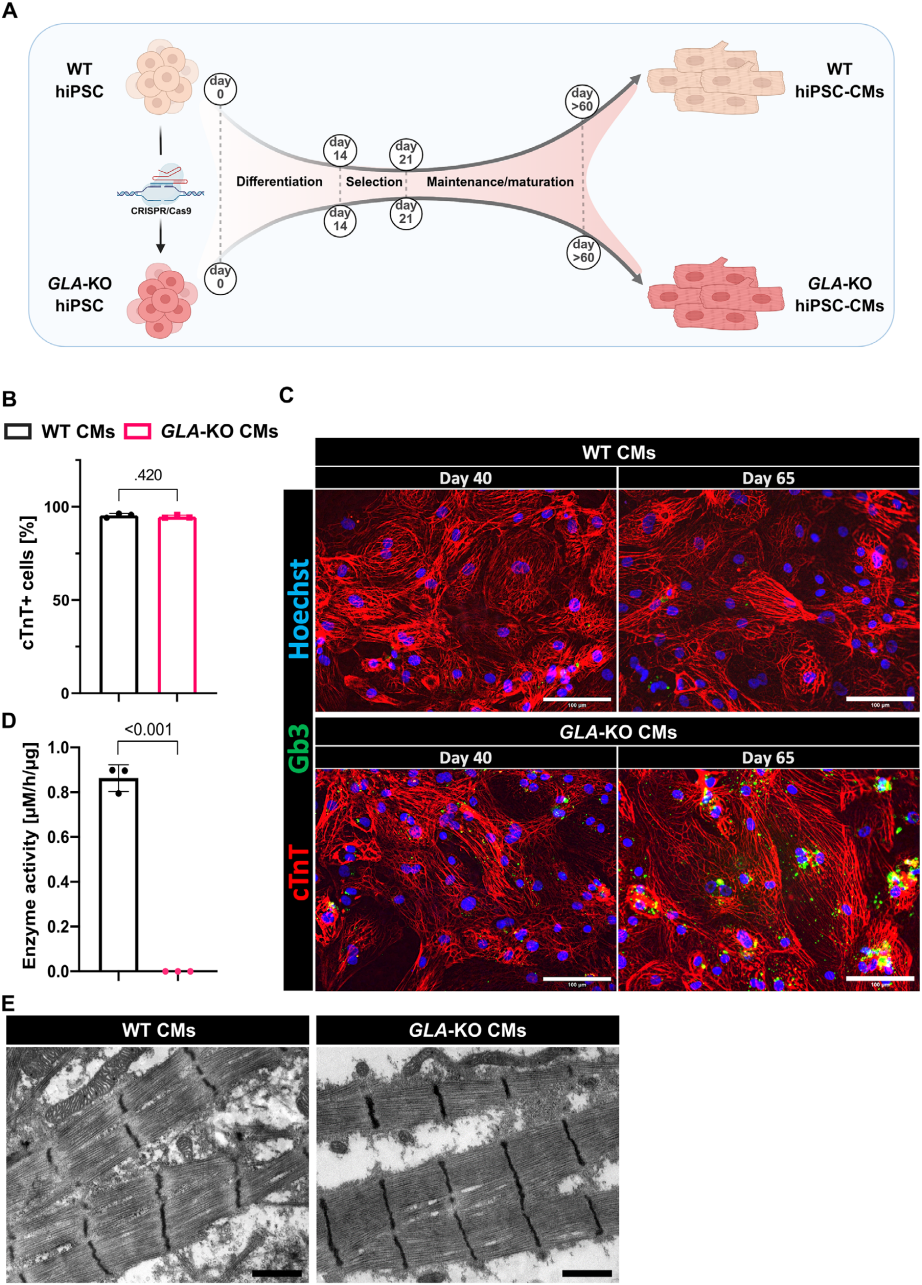
*GLA*‐KO CMs are α‐galactosidase A enzyme deficient and progressively accumulate Gb3. (A) Schematic for the generation of a patient‐independent hiPSC‐derived in vitro model for the cardiac manifestation of Fabry disease. Created in BioRender. Hoepfner, J. (2026) https://BioRender.com/kouwaxh. (B) Cardiac differentiation efficiency as analyzed by flow cytometry for cardiac troponin T (cTnT) positive cells; (n = 3 independent differentiations, mean (SD), unpaired *t*‐test, α = 0.05). (C) Progressive Gb3 accumulation in cTnT‐expressing CMs analyzed in 40‐ and 65‐day‐old CMs by fluorescence microscopy. Scale bars indicate 100 µm. (D) The α‐galactosidase A enzyme activity measured in cell lysates of 65‐days‐old CMs; (n = 3 independent differentiations, unpaired t‐test, α = 0.05). (E) Ultrastructural analysis of CM sarcomeres using transmission electron microscopy. Scale bars indicate 1 µm.

### 2.2 Transcriptome Profiles Resemble Key Phenotypes of Fabry Disease

To ascertain the capacity of present in vitro disease model to replicate the cardiac manifestation of Fabry disease and elucidate its pathomechanism, we performed bulk‐RNA sequencing of rRNA‐depleted total RNA isolated from 65‐day‐old WT and *GLA*‐KO CMs (Figure 2A). When subjecting the subset of 3359 differentially expressed genes (DEGs; base mean > 5; q < 0.1) to enrichment analysis (g:profiler), these DEGs were particularly enriched in pathways previously associated with Fabry disease (Figure 2B). Specifically, pathways related to apoptosis [22], mitochondrial energy metabolism [3], and reactive oxygen species [6] were deregulated. DEGs were also enriched in terms related to the physiological and pathophysiological function of the heart, namely, “cardiac muscle contraction” and “diabetic cardiomyopathy” (Figure 2C). Of potential interest, several signaling pathways, specifically, Ras/MAPK, TGF‐beta and integrin signaling were significantly enriched for the identified DEGs.

**FIGURE 2:**
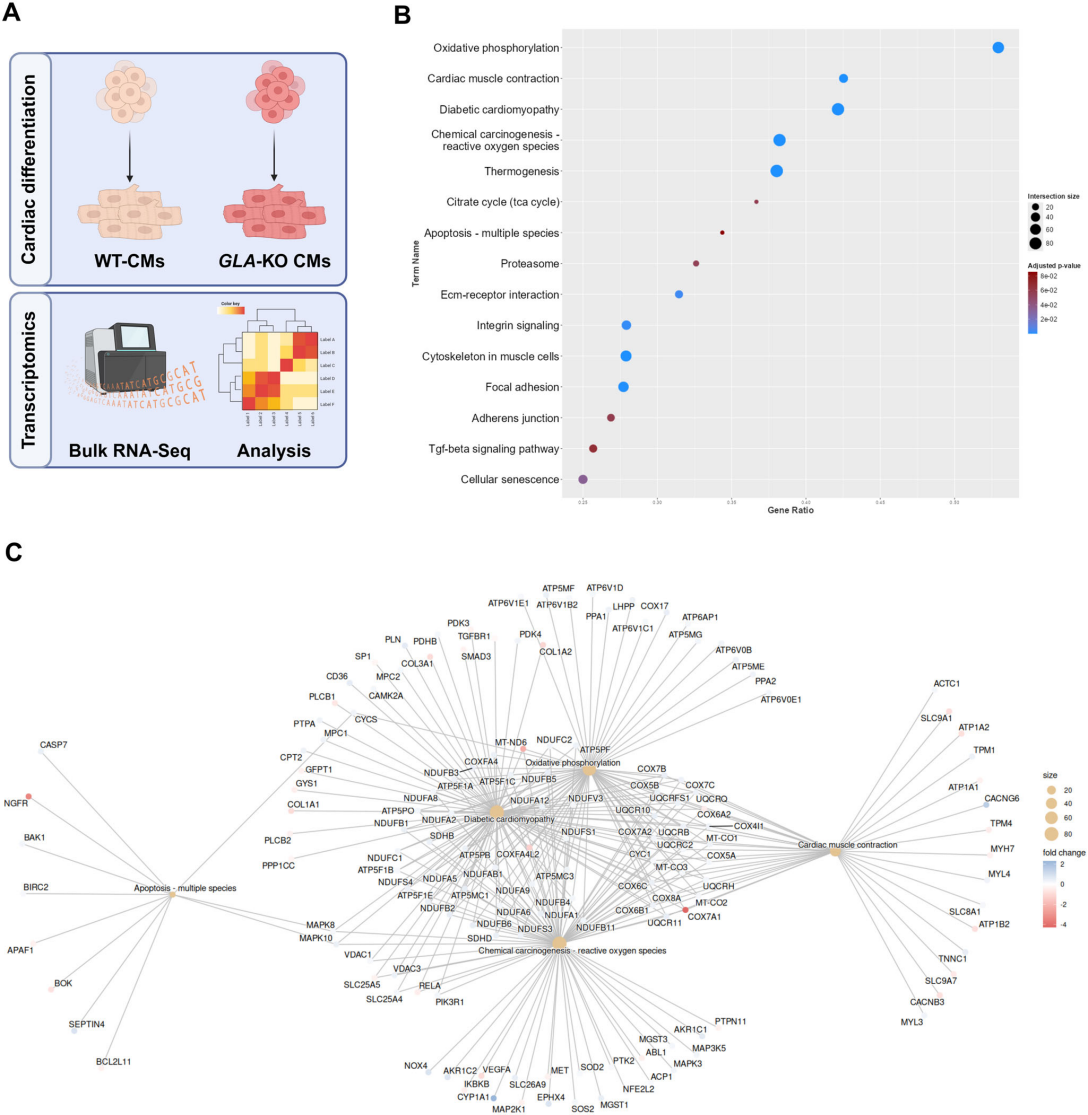
Comparative transcriptomics mirrors key Fabry disease phenotypes. (A) Schematic representation of the transcriptomics approach comparing day‐65‐old WT vs. *GLA*‐KO CMs. Created in BioRender. Hoepfner, J. (2026) https://BioRender.com/85yv3f9. (B) Bubble plot depicting key pathway terms enriched for significantly dysregulated genes between WT and *GLA*‐KO CMs; (*q* ≤ 0.1 and base mean ≥ 5, n = 4 independent differentiations per group). (C) Cnetplot displaying gene‐pathway networks enriched for genes significantly differentially expressed (FDR‐adjusted *p*‐value ≤ 0.1) between *GLA*‐KO and isogenic WT control CMs. Log_2_‐fold changes derived from *DeSeq2* are represented by node colors.

In essence, our transcriptomic approach both reflects well‐established cellular phenotypes of Fabry disease and may provide new avenues of research.

### 2.3 Nucleoside‐Modified α‐Galactosidase A mRNA Therapy Effectively Restores α‐Galactosidase A Enzyme Activity‐ in *GLA*‐KO CMs

The available treatment options insufficiently manage Fabry disease, creating reasons to develop novel therapeutic strategies. A particularly promising modality that is steadily gaining pace and has generated first promising results in preclinical studies in vitro [18] and in vivo both in mice and non‐human primates [17] is the use of *GLA* mRNA. However, as of now it remains unclear, whether *GLA* mRNA holds the potential to effectively address the archetypical cellular Fabry disease phenotypes, as the available animal models poorly reflect them, and in vitro studies are limited.

To address this question, we synthesized modGLA as a potential therapeutic intervention. The RNA was transcribed in vitro from a linearized plasmid template encoding the *GLA* open reading frame flanked by the alpha globin 5’‐untranslated and beta globin 3’‐untranslated region (Figure 3A). All uridines in the modRNA were substituted for with the modified nucleoside N1‐methyl‐pseudouridine (m1ψ), and co‐transcriptional capping was performed utilizing the Cap 1 analogue CleanCap AG. These modifications are known to reduce immunogenicity, while also allowing an enhanced translation efficiency [23, 24].

**FIGURE 3:**
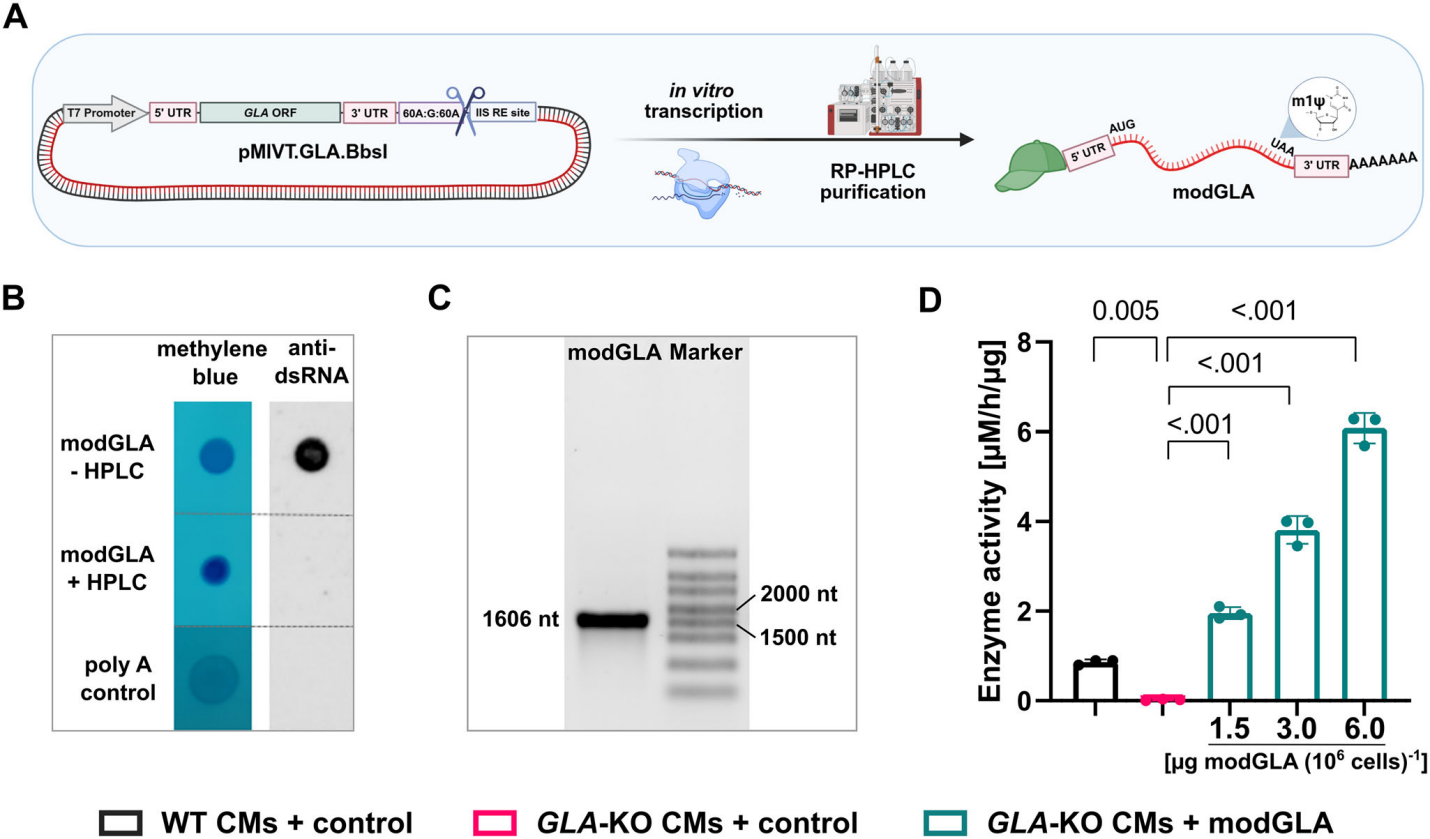
Nucleoside‐modified *GLA* mRNA effectively restores α‐galactosidase A enzyme function. (A) Production pipeline for nucleoside‐modified *GLA* mRNA (modGLA) by in vitro transcription (IVT) from linearized plasmid templates followed by IP‐RP‐HPLC‐based purification. Created in BioRender. Hoepfner, J. (2026) https://BioRender.com/wqdk2xg. (B) Dot Blot validating the successful removal of dsRNA byproducts from modGLA IVT products (modGLA—HPLC) by IP‐RP‐HPLC (modGLA + HPLC). (C) Analytical sodium hypochlorite agarose gel for analysis of purified modGLA. (D) Dose‐dependent rescue of α‐galactosidase A enzyme activity in *GLA*‐KO CMs transfected with modGLA; (n = 3 independent differentiations, mean (SD), ordinary one‐way ANOVA followed by Tukey's multiple comparisons test, α = 0.05).

Subsequently, modGLA was purified using ion‐pair reversed‐phase high performance liquid chromatography (IP‐RP‐HPLC) (Figure 3A) which effectively eliminates dsRNA byproducts [25] commonly found in T7 RNA polymerase transcription products [26] (Figure 3B). Following RNA purification, denaturing sodium hypochlorite gel electrophoresis confirmed modGLA's expected size of 1606 nucleotides, and its integrity (Figure 3C). Finally, transfecting increasing doses of modGLA into *GLA*‐KO CMs dose‐dependently rescued α‐galactosidase A enzyme activity (Figure 3D). In the subsequent experiments, 6 µg modGLA per one million cells were applied since this dose produced the highest increase of α‐galactosidase A enzyme activity without affecting cell viability, as measured by WST assay (Figure S1) while showing a low immunogenic potential to induce an innate immune response when compared to unmodified GLA mRNA (Figure S2).

### 2.4 A‐galactosidase A Enzyme Deficiency Results in a Cellular Fabry Disease Phenotype That Is Significantly Rescued by modGLA Therapy

While the molecular intricacies leading to the multifaceted cardiac manifestation of Fabry disease remain elusive, the trigger appears to be the massive deposition of α‐galactosidase A glycosphingolipid substrates, including Gb3, within patients’ cells [5]. To investigate whether functional rescue of the α‐galactosidase A enzyme activity and reduction of substrate levels is concomitant with a phenotypical rescue, we utilized modGLA as a therapeutic intervention. A weekly dosing regimen was selected based on the kinetics of α‐galactosidase A enzyme activity in response to a single dose of modGLA. Seven days after transfection, enzyme activity had decreased substantially but remained modestly, yet significantly, increased above control levels. Redosing at this time‐point was intended to maintain enzyme activity continuously above baseline, thereby providing sustained enzymatic correction while minimizing dosing frequency (Figure S3). Following the administration of four weekly doses of therapeutic modGLA, or control modRNA (modfLuc, modEGFP, vehicle), the CMs were subjected to functional analyses at approximately 65 (± 5) days of age (Figure 4A). The maturation state of hiPSC‐CMs in this study was contextualized based on previous work employing the same general differentiation approach, which demonstrated that hiPSC‐CMs beyond 50 days post‐differentiation exhibit features of functional maturation, including on the electrophysiological level evidenced by increased I_Ca,L_, I_Na_, and I_K1_ densities, and improved calcium handling properties [27]. While comprehensive maturation assessment was not performed in the present study, the analyses were conducted at day 65 post‐differentiation, a time point within the long‐term culture maturation window characterized in this prior work.

**FIGURE 4:**
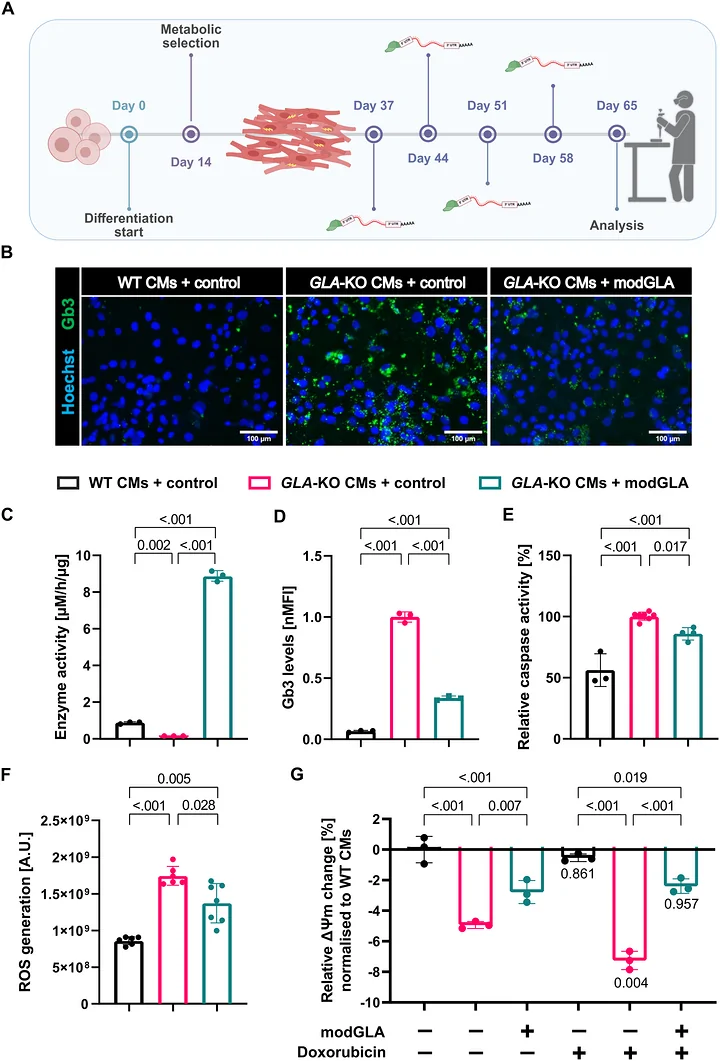
Glycosphingolipid accumulation and cellular disease phenotypes are rescued by modGLA therapy in Fabry cardiomyocytes. (A) Weekly treatment regimen of WT and *GLA*‐KO CMs with modGLA, or control modRNA (modfLuc; modEGFP) or vehicle (only E). Time‐points represent ranges of ± 5 days, while the intervals are consistently 7 days. The analyses in (B – G) were performed one week after the last modGLA dose. Created in BioRender. Bär, C. (20265) https://BioRender.com/x5t35jn. (B) Rescue of Gb3 accumulation in *GLA*‐KO CMs by modGLA treatment as analyzed by fluorescence microscopy. Nuclei are stained by Hoechst33342. Scale bars represent 100 µm. (C) The α‐galactosidase A enzyme activity as measured by α‐galactosidase A enzyme activity assay in CM lysates; (n = 3 independent differentiations, mean (SD), ordinary one‐way ANOVA followed by Tukey's multiple comparisons test, α = 0.05). (D) Quantification of Gb3 signal in CMs from fluorescence microscopic images as those depicted in B, normalized to the number of nuclei, and expressed as “normalized mean fluorescence intensity (nMFI); (n = 3 independent differentiations, mean (SD), ordinary one‐way ANOVA followed by Tukey's multiple comparisons test, α = 0.05). (E) Relative caspase 3/7 activity measured by caspase‐Glo 3/7 assay in CMs challenged for 18 h with 2.56 µm doxorubicin; (n = 3–6 independent differentiations, mean (SD), ordinary one‐way ANOVA followed by Tukey's multiple comparisons test, α = 0.05). (F) Generation of reactive oxygen species (ROS) over a period of 6 h in CMs challenged with 100 µm H_2_O_2_ as measured by DCFDA assay in arbitrary units (A.U.); (n = 6–7 independent differentiations, mean (SD), Brown‐Forsythe and Welch's ANOVA followed by Dunnett's T3 multiple comparisons test, α = 0.05). (G) Mitochondrial membrane potential (∆ψm) relatively quantified in CMs with or without prior challenge with 1 µm doxorubicin for 24 h; (n = 3 independent differentiations, mean (SD), two‐way ANOVA followed by Tukey's multiple comparisons test, *p*‐values below bars represent comparisons between means of equal color code, α = 0.05).

At this stage, we focused on well‐established cellular phenotypes of Fabry disease that were also reflected in the earlier transcriptome analysis such as apoptosis, oxidative stress, and mitochondrial dysfunction. Evidently, modGLA therapy significantly rescued α‐galactosidase A enzyme deficiency in *GLA*‐KO CMs (Figure 4C). Automated fluorescence microscopic analysis of Gb3‐associated signal revealed that this restored enzyme function significantly reduced Gb3 deposition within these cells, decreasing by on average 66.4% compared to modfLuc control‐treated *GLA*‐KO CMs (Figure 4B, D). In a second approach, we applied only a single dose of modGLA or modEGFP to 58‐day‐old CMs and quantified glycosphingolipid levels one‐week post‐treatment by multiplex capillary gel electrophoresis coupled to laser‐induced fluorescence detection (xCGE‐LIF). Peak intensities were normalized to a defined, spiked‐in mannose 6 glycan standard, enabling direct inter‐sample quantitative comparisons (detection limit of approximately 10 pg per glycan). This quantitative approach confirmed that modGLA treatment significantly rescued altered glycosphingolipid levels, specifically Gb3, galactobiosylceramide and nLc6 (Figure S4).

To assess the sensitivity of CMs to apoptotic stimuli, the cells were challenged with the cardiotoxic anthracycline doxorubicin prior to analysis. Strikingly, the changes observed in Gb3 levels were reflected in caspase 3/7 activities. Specifically, in comparison to WT CMs, control‐treated diseased CMs exhibited significantly higher relative caspase 3/7 activity in response to the stress stimulus, which normalized significantly by approximately 32% in modGLA‐treated *GLA*‐KO CMs (Figure 4E). Additionally, we investigated the antioxidative capacity of our Fabry disease model by measuring the production of reactive oxygen species (ROS) in response to oxidative stress in form of hydrogen peroxide via H2DCFDA assay. As with the elevated caspase activity, control *GLA*‐KO CMs also exhibited the greatest increase in ROS levels, which again normalized significantly by on average 41.8% upon modGLA treatment (Figure 4F). Finally, we used the fluorescent dye tetramethyl rhodamine ethyl ester (TMRE) in conjunction with flow cytometry to indirectly measure the mitochondrial membrane potential (∆ψm) of CMs (Figure 4G). Interestingly, already in unchallenged CMs we observed a significant depolarizing shift in the ∆ψm in control *GLA*‐KO CMs relative to control WT CMs that was partly rescued by modGLA treatment. However, after the cells were pre‐treated for 24 h with 1 µm doxorubicin, we observed this depolarizing shift to worsen in the control *GLA*‐KO CMs. Such change was not observed in the control WT CMs or the modGLA‐treated diseased CMs.

In summary, Fabry‐archetypical phenotypes were confirmed at a functional level in our in vitro model, and for the first time, we demonstrated their rescue upon modGLA treatment.

### 2.5 *GLA*‐KO CMs Display Accelerated Decay Parameters of Calcium Transients and Increased Phospholamban Phosphorylation

Pathway enrichment analysis performed with the significantly DEGs between *GLA*‐KO and WT CMs, identified “cardiac muscle contraction” as a potentially relevant KEGG pathway. Because myocardial contraction is largely controlled by the spatio‐temporal regulation of calcium ion concentrations [28], we performed calcium imaging using the ratio metric calcium sensor Fura2‐AM. The analysis of recorded calcium transients revealed a significantly increased decay velocity concurrent with a decreased 50% decay time in the diseased CMs, which normalized upon modGLA treatment (Figure 5A, B). A similar trend was observed with 7.9% of *GLA*‐KO CMs displaying arrhythmic calcium transients, dropping to only 1.7% of cells following modGLA therapy (Figure 5C, Figure S5). The calcium decay parameters are indicative of the reduction phase of temporarily higher cytoplasmic calcium concentrations during cardiac excitation‐contraction coupling (Figure 5D). In addition, we detected an increased caffeine‐induced calcium amplitude in Fabry CMs (Figure S6). This led us to hypothesize a gain‐of‐function in the mechanisms shuttling calcium ions either out of the cell (NCX1) or back into the sarcoplasmic reticulum (via SERCA2a, regulated by its reversible inhibitor PLN). Strikingly, we observed no significant changes in the protein levels of NCX1 or SERCA2a between the disease model and respective controls (Figure 5E–G). However, the relative levels of PLN commonly phosphorylated by cAMP‐dependent protein kinase A (PKA) at serine 16 (pSer16‐PLN), were significantly higher in the diseased CMs (Figure 5H). This post‐translational modification is known to relieve PLN's inhibitory effect on SERCA2 [29]. Notably, the hyperphosphorylation of PLN normalized in response to modGLA therapy. Interestingly, we observed globally comparable phosphorylation levels of PKA substrates (p‐PKA), between controls, and modGLA treated *GLA*‐KO CMs. However, the disease model displayed a decreased responsiveness to PKA inhibition (PKI), while its’ response to protein phosphatase inhibition (Calyculin A) was increased, which normalized after modGLA therapy (Figure S7).

**FIGURE 5:**
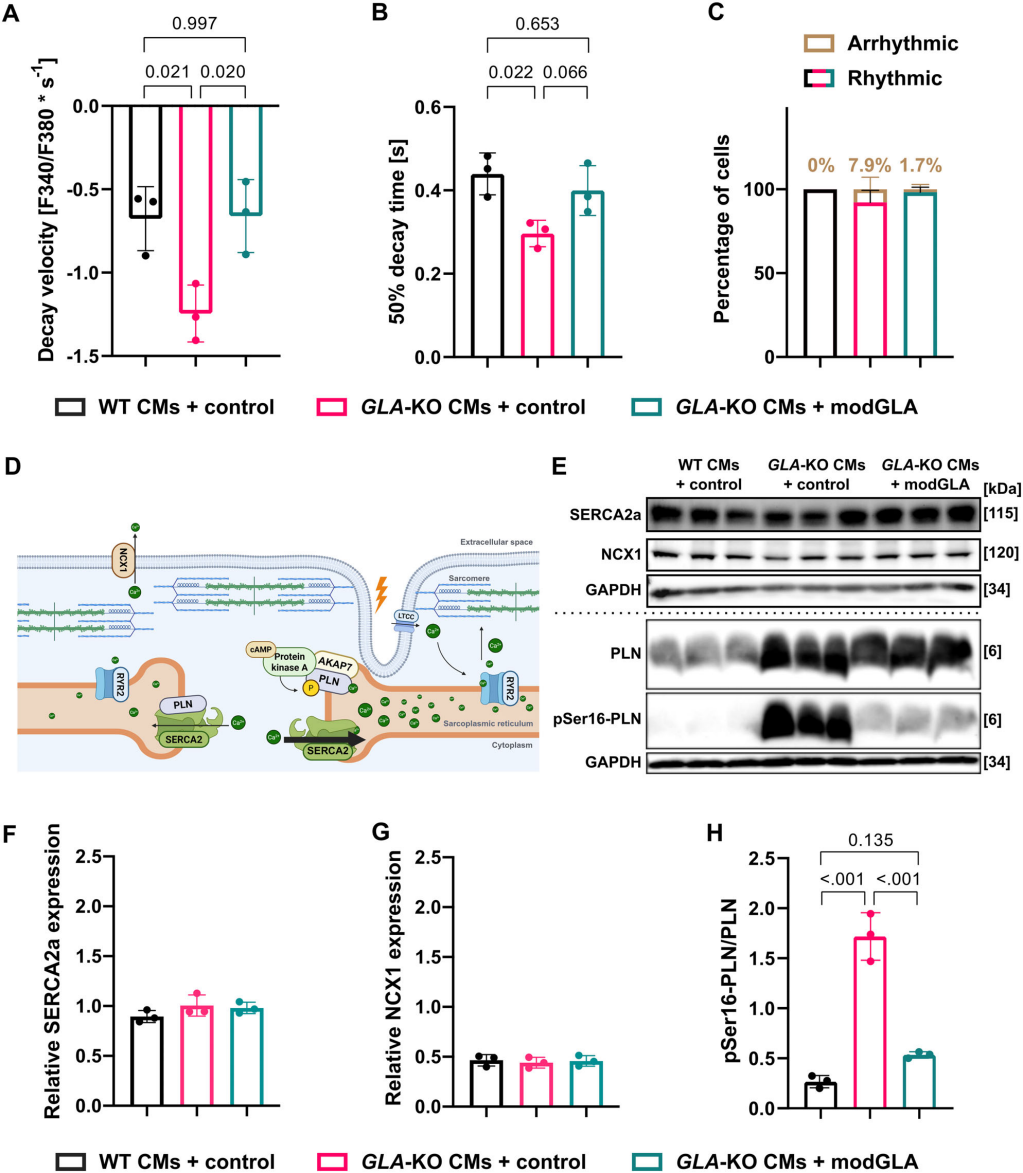
Enhanced calcium handling and hyperphosphorylated PLN in Fabry cardiomyocytes is partly normalized by modGLA therapy. Calcium imaging in CMs performed using the ratio metric calcium indicator Fura 2 to determine the (A) decay velocity of calcium transients as analyzed by the change in the ratio metric Fura 2 fluorescence in seconds [F340/380^*^s^−1^] and (B) the 50% decay time [s]; (n = 3 independent differentiations, calcium transients of 18–39 cells per replicate and group were recorded, mean (SD), ordinary one‐way ANOVA followed by Tukey's multiple comparisons test, α = 0.05; performed on log‐transformed data after adding an offset constant if values were previously negative). (C) Fraction of cells [%] showing arrhythmic calcium transients during calcium imaging; (n = 3 independent differentiations, calcium transients of 18–39 cells per replicate and group were recorded). (D) Schematic depiction of cardiac excitation‐contraction coupling and the fine‐tuning of calcium dynamics by the reversible SERCA2a‐inhibitor phospholamban (PLN). Created in BioRender. Bär, C. (2026) https://BioRender.com/dc4kxkr. (E) Protein levels of SERCA2a, NCX1, total PLN, and PLN phosphorylated at serine 16 (pSer16‐PLN) relative to GAPDH, analyzed by Western blot. The dotted line indicates two individual membranes. After detecting SERCA2a or pSer16‐PLN, the respective membrane was stripped and reprobed for NCX1 or PLN, respectively. (F—H). Densitometric quantification of (F) SERCA2a, (G) NCX1, as well as (H) pSer16‐PLN over total PLN; relative to GAPDH detected on the corresponding membranes depicted in (E); (n = 3 independent differentiations, ordinary one‐way ANOVA followed by Tukey's multiple comparisons test; α = 0.05).

Furthermore, we observed decreased trends of 90% decay time, decay rate τ and diastolic calcium in the Fabry CMs (Figure S5A, B, D). In contrast, the maximum rise velocity and calcium amplitude appeared to be increased in the disease model (see Figure S5C, E). Interestingly, the described alterations normalized upon modGLA treatment.

### 2.6 Comparative Transcriptomics in Fabry Patient‐Derived CMs Identifies Calcium Signaling as a Driver of Cardiac Manifestations

To validate the key findings observed in our generalized, patient‐independent Fabry disease model in a disease‐relevant, patient‐specific context, we next employed an additional hiPSC‐based in vitro model (F01 hiPSC), derived from a male Fabry patient carrying the c.959A > T missense mutation in *GLA* [30]. The corresponding isogenic control hiPSC line (F01‐IC) was generated by prime editing CRISPR/Cas technology. Specifically, we differentiated all hiPSCs into CMs as described before and subjected both, the patient‐derived (F01 vs. F01‐IC CMs) and patient‐independent CMs (*GLA*‐KO vs. WT CMs), to bulk‐RNA sequencing to compare the transcriptional profile of the two models (Figure 6A). This approach allowed us to assess, which molecular changes could be Fabry‐distinctive, by focusing particularly on those DEGs with directionally similar expression changes (highlighted in green in Figure 6B; reciprocally regulated transcripts are marked red). Strikingly, when performing enrichment analysis on this subset of DEGs, calcium signaling was amongst the significantly enriched pathways (Figure 6C). Additionally, in accordance with the initial pathway analysis (see Figure 2B, C), DEGs with concordant regulation patterns between the two models were enriched in the term cardiac muscle contraction, as well as in pathways related to ROS, metabolism, ECM‐receptor interactions, and programmed cell death (more detailed pathway analysis and gene‐pathway networks, both model‐specific and Fabry‐distinctive across models are depicted in Figures S8–S10). In addition, as calcium signaling and cardiac muscle contraction are intimately linked, we assessed contractility in patient independent and derived Fabry CMs. Consistent across models we detected accelerated contraction times (10–10 and 50–50) and decreased contraction amplitudes. These contractile alterations were partly restored by modGLA therapy (Figure S11). On protein level, and consistent with our initial results in patient‐independent *GLA*‐KO CMs, we found PLN to be hyperphosphorylated at serine 16 in patient‐derived F01 CMs. Strikingly, this observation was partially reversed upon modGLA treatment (Figure 6D‐G). In a more translational angle, modGLA was encapsulated by microfluidic mixing into a lipid nanoparticle (LNP) formulation, which achieved >90% transfection efficiency in hiPSC‐CMs (Figure S12), and increased α‐galactosidase A enzyme activity consistently in both adult porcine cardiac tissue in form of living myocardial slices (LMS) and human precision‐cut liver slices (hPCLiS) (Figure S13). Importantly, modGLA‐LNPs also rescued this primary disease phenotype and reduced Gb3 deposition in disease‐relevant patient‐derived Fabry hiPSC‐CMs and hiPSC‐ endothelial cells (ECs) (Figure S14).

**FIGURE 6:**
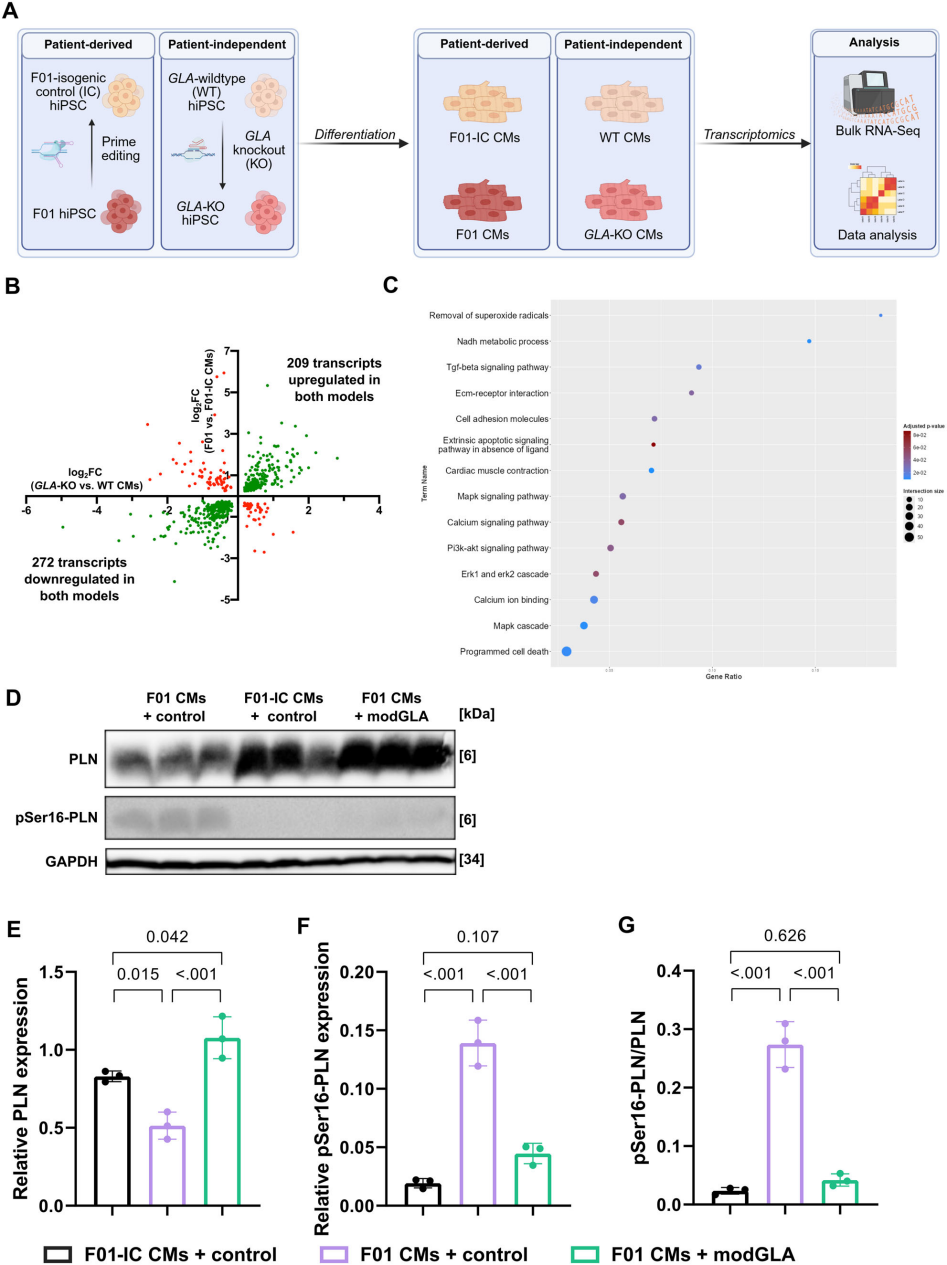
Comparative transcriptomics unravels Fabry‐distinctive pathway signature. (A) Illustration of the combined approach applied to compare the transcriptional profile of two independent Fabry disease models, one patient‐derived (F01 (n = 3 independent differentiations) vs. F01‐IC CMs (n = 4 independent differentiations)), the other patient‐independent (*GLA*‐KO CMs vs WT CMs, n = 4 independent differentiations). Created in BioRender. Hoepfner, J. (2026) https://BioRender.com/85yv3f9. (B) Cartesian plot depicting the significantly differentially expressed genes (base mean ≥ 5; *q* ≤ 0.1) of two bulk‐RNA sequencing data sets comparing F01 vs. F01‐IC CMs (y‐axis) and *GLA*‐KO vs WT CMs (*x*‐axis), respectively. Directionally similar regulated transcripts are highlighted in green, those transcripts reciprocally regulated between the disease models are labelled red. (C) Bubble plot depicting pathway enrichment analysis of overlapping DEGs that were significantly dysregulated in both disease models (*GLA*‐KO vs. WT cardiomyocytes, and between F01 patient‐derived and isogenic F01‐IC cardiomyocytes; baseMean > 5, *q* < 0.1) and showed concordant direction of change. Enrichment analysis was performed on this shared gene set, and pathways passing an FDR‐adjusted significance threshold of *q* < 0.1 are shown. Bubble size represents the intersection size (number of genes from the input DEG set mapping to each pathway), and bubble color reflects the adjusted *p* value. (D) Total PLN and pSer16‐PLN protein levels as analyzed by Western Blot relative to GAPDH, detected on the same membrane. (E–G) Densitometric quantification of the protein levels detected in (D) relative to GAPDH; (n = 3 independent differentiations, ordinary one‐way ANOVA followed by Tukey's multiple comparisons test; α = 0.05).

In conclusion, by employing an independent patient‐derived model of Fabry cardiomyopathy, we validated the identified altered calcium handling, accompanied by PLN hyperphosphorylation, as a Fabry distinctive phenotype, and replicated therapeutic potential in a second disease model.

## 3 Discussion

Here we report that loss of *GLA* entails several cellular disease phenotypes in hiPSC‐derived CMs previously associated with Fabry disease, highlighting the validity of our disease model. Specifically, we observed an increased sensitivity to pro‐apoptotic stimuli, an impaired capacity to neutralize reactive oxygen species, as well as an impaired mitochondrial membrane potential that deteriorated upon doxorubicin stress. In Fabry patients, glycosphingolipid build‐up is proposed to activate secondary metabolic processes, which over time can manifest as Fabry cardiomyopathy [5]. The increased sensitivity of Fabry CMs to stress stimuli such as doxorubicin and H_2_O_2_ demonstrated in this study, suggests that the underlying cellular dysfunction in Fabry‐affected cells might increase their susceptibility to deleterious factors encountered throughout each patient's lifetime—particularly during periods of infection, inflammation, or the exposure to dietary or pharmacological toxins. Consequently, the ill‐defined genotype‐phenotype correlation in Fabry disease [31], may in part be a result of non‐genetic factors such as the unique lifestyle of each patient, which includes varying levels of these stressors. Future longitudinal and prospective cohort studies could aid in investigating this hypothesis. In addition, the great phenotypical heterogeneity of Fabry disease patients [31], could potentially be deciphered by identifying putative confounding genetic variants by exome sequencing.

Enhanced calcium handling, in particular an increased amplitude and rising slope, as well as an elevated calcium load in the sarcoplasmic reticulum, had been reported before by Birket and colleagues in Fabry hiPSC‐CMs [32]. Similarly, we observed enhanced calcium decay parameters in *GLA*‐KO CMs, as well as an increased caffeine‐induced calcium amplitude in both established models. However, a mechanistic explanation for these observations was lacking thus far. Here we propose a first explanation for these calcium handling abnormalities in form of PLN hyperphosphorylated by PKA. Importantly, SERCA2a activity is tightly regulated by PLN, its reversible inhibitor. This inhibitory effect can be relieved by post‐translational modifications, including phosphorylation at serin 16 by PKA [29]. Consequently, the consistently higher proportion of phosphorylated PLN over total PLN in these models, suggests a disinhibition of PLN's inhibitory effect on SERCA2a function. Functionally, pharmacological investigation of PKA signaling indicated altered phosphorylation dynamics in Fabry CMs, including decreased responsiveness to PKI and increased responsiveness to protein phosphatase inhibition at the global level of p‐PKA substrates, which trended toward normalization after correcting the primary defect by modGLA treatment. In addition, global p‐PKA levels were unaffected by disruption of PKA‐anchoring protein interactions crucial in functional microdomains. In summary, this pattern is compatible with altered compartmentation of the PLN‐SERCA‐PKA signaling axis rather than simple changes in global PKA activity. In result, higher activity of SERCA2a in Fabry CMs likely entails a faster removal of calcium ions from the cytoplasm back into the sarcoplasmic reticulum during cardiac excitation‐contraction coupling. This mechanism is in line with the increased calcium load in the sarcoplasmic reticulum observed by Birket and colleagues [32] and confirmed by us in two independent Fabry CM models. Congruently, we observed trends of lower diastolic calcium concentrations and higher calcium transient amplitudes in *GLA*‐KO CMs (see Figure S5D, E). Unraveling the upstream pathways leading to the increased phosphorylation of PLN's Ser16 residue shall be a focus of future research efforts. Factoring in that the transcriptomics approach comparing the two independent disease models highlighted PI3K‐Akt and Ras/MAPK signaling, two pathways that are known to modulate cardiac calcium homeostasis (e.g [33, 34, 35]), investigations into their molecular interplay in the context of Fabry cardiomyopathy might prove particularly productive. The comparative pathway analysis also highlighted calcium signaling in general, substantiating an important role in the cardiac manifestation of Fabry disease. As to what degree this is the case remains to be answered by future studies.

Huurne et al. have recently demonstrated that *GLA* mRNA can restore α‐galactosidase A enzyme activity in Fabry hiPSC‐CMs and reduce Gb3 accumulation with an RNA dosing regimen every other day from 14 until 21 days post start of differentiation [18]. To further extend these findings, we utilized more mature hiPSC‐CMs, as these cells are reported to develop substantial expression of key ionic currents and hence electrophysiological and calcium handling ability only after prolonged culture of >50 days [27]. Accordingly, we performed the functional analyses 65 days (± 5 days) after differentiation onset. To this date, it remained unknown whether modGLA therapy has the potential to clear Gb3 accumulation from CMs at that age. Notably, by successfully employing modGLA both as a weekly therapeutic intervention and a single dose treatment in relatively mature *GLA*‐KO CMs, we provided first proof that glycosphingolipid deposition can be effectively reduced in CMs even after it manifested substantially. Since diagnosis in patients often comes late after glycosphingolipid accumulation and resulting symptoms already became evident [36], this observation may be of clinical relevance. The effective weekly dosing regimen used in our study further corroborates the translational potential of this approach. However, whether modGLA proves more effective compared to ERT in eliminating substrate deposition from cardiomyocytes in vivo remains to be determined. Nevertheless, the consistent partial restoration of investigated disease phenotypes in response to modGLA therapy confirms their causative relationship with α‐galactosidase A enzyme deficiency and Fabry disease. Additionally, the translational relevance of modGLA therapy is supported by validation in human PCLiS and porcine adult cardiac tissue (LMS), where LNP‐encapsulated modGLA consistently increased α‐galactosidase A enzyme activity.

The partial rescue of some phenotypes might be explained by an incomplete removal of Gb3 deposition from the CMs. While longer treatment periods might clear the residual Gb3, it is conceivable that lysosomes packed with Gb3 become dysfunctional to a degree that might hinder their removal by the autophagic‐lysosomal system or trafficking of functional α‐galactosidase A into these compartments. Aptly, an impaired autophagic‐lysosomal function has been described before by Song and colleagues in a model of Fabry cardiomyopathy [22].

Expanding on the investigations in present study, future studies could increase the total treatment duration to elucidate whether glycosphingolipid deposits can be entirely cleared from affected cells, even when treatment is started after glycosphingolipid accumulation has manifested already. Conversely, more frequent dosing regimens may offer limited benefits, since systemic delivery of for instance lipid nanoparticle‐encapsulated modGLA via intravenous injections several times a week, is not conducive to clinical translation.

Newer approaches such as engineered circular coding RNA [37] might be explored instead to increase the half‐life of the therapeutic RNA and hence reduce dosing frequency, which would not only limit costs but might additionally aid patient compliance.

However, it is conceivable that irreversible cellular damage may have manifested by the time treatment is initiated, which cannot be ameliorated by complete substrate removal alone. Such long lasting changes could for instance be epigenetic changes to patient DNA, as have been reported before [38]. In this case additional therapeutic strategies need to be developed that specifically target these altered pathways.

In summary, we provide mechanistic insight into the early cellular manifestation of Fabry cardiomyopathy, particularly in form of abnormal calcium handling, likely impacted by PLN hyperphosphorylation. In addition, our data illustrate that mRNA therapy can rescue archetypical cellular disease phenotypes in Fabry hiPSC‐CMs, supporting its potential as a future therapeutic strategy for Fabry disease.

## 4 Material and Methods

### 4.1 Maintenance and Differentiation of hiPSCs

HiPSCs were maintained in E8 medium (as described in Chen et al. [39]: DMEM F12 HEPES, 543 mg/l NaHCO_3_, 20 mg/l insulin, 10.7 mg/l holo‐transferrin, 2 µg/l TGFβ1, 64 mg/l ascorbic acid 2‐phosphate, 14 µg/l sodium selenite, 20 µg/l bFGF) and grown to 90%–100% confluency on GelTrex‐coated (25 µg/cm^2^) cell culture plates before passaging every 4–5 days into E8 medium supplemented with 2 µm Thiazovivin using 480 mm EDTA in DPBS. Differentiation into CMs was performed as described before [40] by passaging the hiPSCs in clusters and subjecting them to biphasic Wnt pathway modulation (in RPMI1640 and GlutaMAX with HEPES, 0.5% (w/v) recombinant human albumin, 0.2% (w/v) L‐ascorbic acid 2‐phosphate sesquimagnesium salt hydrate: two days supplemented with CHIR99021, two consecutive days supplemented with IWP‐2, and finally 4 days medium only). Eight days after start of differentiation, spontaneous contractions started and medium was switched to RPMI1640 and GlutaMAX with HEPES, 2% (w/v) serum‐free B27 supplement for one week before performing metabolic selection in RPMI1640 without glucose, 0.5% (w/v) recombinant human albumin, 0.2% (w/v) L‐ascorbic acid 2‐phosphate sesquimagnesium salt hydrate, 4 mm for another week. Selected hiPSC‐CMs were maintained in RPMI1640 and GlutaMAX with HEPES, 2% (w/v) serum‐free B27 supplement. HiPSCs were differentiated into endothelial cells (ECs) using a suspension‐based protocol. Briefly, single‐cell hiPSC suspensions were seeded in uncoated 6‐well plates and cultured overnight in E8 medium supplemented with thiazovivin under orbital shaking (70 rpm). Mesoderm induction was initiated by treatment with N2B27 medium containing CHIR99021 (7.5 µm) and BMP4 (25 ng mL^−^^1^) for 3 days, with medium refreshment on day 3. On day 4, cells were switched to StemPro‐34 medium supplemented with VEGF165 (200 ng mL^−^^1^) and forskolin (2 µm) to promote endothelial specification. On day 6, differentiated cultures were dissociated using collagenase II and Accutase, and CD31^+^ ECs were enriched by magnetic‐activated cell sorting (MACS; Miltenyi Biotec) using anti‐CD31 microbeads. Endothelial purity was assessed by flow cytometry using APC‐conjugated anti‐human CD31 antibodies. Purified ECs were either cryopreserved or cultured on Geltrex‐coated cell culture vessels in Endothelial Growth Medium‐2 (EGM‐2, Lonza), with medium replacement every other day.

### 4.2 Alpha‐galactosidase Enzyme Activity Assay

The α‐galactosidase A enzyme activity was determined as described before [41], with few modifications. In brief, cell lysates were prepared for 30 min at 4°C on a rotating device using basal buffer (46 mm sodium phosphate dibasic, 27 mm sodium citrate, pH 4.6) with 0.5% Triton X‐100. The lysates (20 µl) were incubated in black microtiter plates with 40 µl assay buffer (basal buffer with 117 mm N‐acetyl‐D‐galactosamine and 6 mm 4‐methylumbelliferyl‐α‐D‐galactopyranoside) at 37°C for 1 h. Next, the reaction was stopped by adding 70 µl of 400 mm glycine at pH 10.8. After adding a 4‐methylumbelliferone standard (0.5 – 100 µm) to the plate, the fluorescence was detected using a Synergy HT plate reader (365/488 nm, Agilent Technologies). The α‐galactosidase A enzyme activity was calculated from the standard curve and the known protein concentration of samples determined by Pierce BCA protein assay kit, as µm of 4‐methylumbelliferone/h/µg of protein.

### 4.3 Immunocytochemistry

Following a wash in DPBS, cultured cells were fixed in 4% PFA for 15 min at room temperature (RT). Permeabilization was performed for 20 min at RT (DPBS + 0.5% Tween‐20, 0.1% Triton X‐100, 0.1% Igepal CA‐630), before staining the cells overnight at 4°C with the respective primary antibodies (Gb3: 1:500, #A2506, TCI; cTnT: 1:400, #ab45932, Abcam; CD31: 1:200, #PA516301, Thermo Fisher Scientific) diluted in DPBS + 1% BSA + 0.3% Triton X‐100. The following day, staining with the secondary antibodies (Anti‐mouse IgG Alexa Fluor 488: 1:500, #A‐21202; Anti‐rabbit IgG Alexa Fluor 594: 1:500, #A‐21207; Thermo Fisher Scientific) and Hoechst33342 (1:2,000) was performed for 1 h at RT. Microplates were imaged using a Cytation 1 device, while glass slides were first mounted in Vectashield HardSetTM Antifade Mounting Medium and then imaged on a Keyence BZ‐X810 fluorescence microscope.

### 4.4 Flow Cytometry

To determine the differentiation efficiency of hiPSCs into derived CMs, flow cytometry was utilized. In brief, hiPSC‐CMs were dissociated into single cells using 0.25X Trypsin/EDTA for 5 min at 37°C, then pelleted at 300 x g for 5 min and fixed in 4% PFA for 20 min at RT. Following two washing steps in DPBS, 10^6^ cells were permeabilized (DPBS + 1% BSA + 0.1% Triton X‐100) per sample. Staining was performed for 1 h at RT with either isotype control (REA Control Antibody, human IgG1, FITC, REAfinity; #130‐113‐449, Miltenyi) or anti cardiac troponin T antibody (Cardiac Troponin T Antibody, anti‐human/mouse/rat, FITC, REAfinity; #130‐119‐674, Miltenyi) diluted 1:50 in DPBS + 1% BSA + 0.1% Triton X‐100. Following two washes, cells were pelleted and resuspended in DPBS + 1% BSA + 2 mM EDTA to approximately 10^6^ cells/ml. Analysis was performed using a CytoFLEX S flow cytometer (Ex/Em: 488/517 nm).

### 4.5 Transmission Electron Microscopy

Cells were grown on membranes in well inserts (Greiner Bio‐One 657651, 0.4 µm pore size thin certs) and fixed by submersion in 150 mm HEPES buffer at pH 7.35 containing 1.5% formaldehyde and 1.5% glutaraldehyde at RT for 30 min and then over night at 4°C. The membrane was cut off the insert and postfixed and stained in 1% osmium tetroxide for 2 h at RT and then over night at 4°C in 1% uranyl acetate, both aqueous solutions. After dehydration in acetone, samples were embedded in EPON and polymerized between aclar foils. The foil was removed afterward, and the samples glued on EPON blocks and cut parallel to the cells. 50 nm sections were poststained with uranyl acetate and lead citrate [42] and observed in a Zeiss EM 900 equipped with a side mounted camera (TRS, Moorenweis).

### 4.6 Transcriptome Analysis

To perform bulk‐RNA sequencing, total RNA was extracted using the miRNeasy mini kit (Qiagen) from hiPSC‐CMs according to the manufacturer's instructions for RNA isolation from cultured cells. To validate a comparable RNA quality of samples, the RNA integrity was determined using a Nano 6000 kit on a Bioanalyzer 2100 (Agilent Technologies). The RNA sequencing and preceding directional library preparation was performed by NovoGene Co. In brief, total RNA (500 ng per sample) was rRNA depleted and then isolated using ethanol precipitation. Following fragmentation, first strand cDNA synthesis was performed using random hexamer primers. Second strand cDNA synthesis was performed while substituting dUTPs for dTTPs. Next, A‐tailing was utilized and after that adapter ligation, size selection, USER enzyme digestion and amplification followed by purification to finalize the library. Then, quality control of the library was performed using a Qubit 2.0 (Thermo Fisher Scientific), a Bioanalyzer 2100 and real‐time PCR. Using an Illumina Novaseq 6000 device in conjunction with an S4 flow cell and v1.5 reagent, the pooled libraries were sequenced with a paired‐end strategy (PE150).

The processing and analysis of raw read data was performed using *hisat2* for alignment against the human reference genome (Ensembl release 114). Next, *feature counts* was used to quantify raw read counts. For differential gene expression analysis, the R package *DESeq2* was used with default settings. Finally, pathway analysis was performed using *g:profiler* with the statistical domain scope set to only annotated genes. Bubble plots of selected enriched pathways (Benjamini‐Hochberg FDR‐corrected *q* < 0.1) were plotted using the R package ggplot2. Gene‐Pathway network plots were generated using the *cnetplot* function from the *clusterprofiler* R package.

### 4.7 Gene Correction of Fabry Patient‐Derived Human iPSCs Using Prime Editing

*GLA* gene correction of the human iPSC line “MHHi029‐A [30]” carrying the c.959A>T mutation, was performed using prime editing, based on transient transfection of the PB‐PE plasmid system using Lipofectamine Stem (Thermo Fisher Scientific), as described elsewhere [43]. Specifically, pegRNA oligonucleotides were annealed and cloned into the BbsI‐HF/BsaI‐HFv2‐linearized PB‐PE backbone. The pegRNA consisted of the scaffold sequence (see Eggenschwiler et al. [43]), the reverse transcriptase template (CATCAATCAGGACCCGTTGG; changes compared to target sequence underlined), and the pegRNA primer binding site (GCAAGCAAGGGTA).

### 4.8 Production of Nucleoside‐Modified mRNA (modRNA)

Nucleoside‐modified mRNA encoding for firefly luciferase (modfLuc), human α‐galactosidase A (modGLA) or enhanced green fluorescent protein (modEGFP) were produced by in vitro transcription. First, specific plasmid templates were generated from pMA‐RQ backbones each encoding: CleanCap AG‐compatible T7‐promoter—human α‐globin 5’ UTR – KOZAK – ORF of the gene of interest—human β‐globin 3’ UTR – segmented poly adenine stretch (60A:G:60A; see Trepotec et al. [44].) – type II S restriction enzyme site (BspQI/BbsI/BtgzI). These plasmid templates (see supplemental information for sequence information) were linearized by restriction digestion and purified by general phenol/chloroform extraction before being transcribed in vitro into modRNA (for a 1 mL in vitro transcription reaction: 50 µg linearized pDNA, 8000 U T7 RNA polymerase, 1000 U RNase inhibitor, 2 U inorganic pyrophosphatase, 4 mm CleanCap AG, 5 mm each of ATP, CTP, GTP and m1ψ, 4 mm Tris‐HCl pH 8, 10 mm DTT, 2 mm spermidine, 0.002% Triton X‐100, 16.5 mm magnesium acetate) at 37°C for 4–24 h. For production of unmodified mRNA m1ψ was substituted with UTP. DNA templates were removed by TURBO DNase digestion (100 U/ml IVT reaction; 15 min at 37°C). The modRNA was ultrafiltrated with Amicon Ultra‐4 centrifugal filters (10 kDa MWCO) before removing 5’‐triphosphates by Antarctic phosphatase treatment (5 U/pmole of uncapped RNA ends assuming 95% capping efficiency; 30 min at 37°C). Purification was performed via IP‐RP‐HPLC on an Äkta pure 25 device in conjunction with a PLRP‐S column at 62°C (Agilent Technologies: column volume (CV) = 4.16 mL; 4000 Å; 30 µm); or using the Monarch RNA Cleanup Kit (NEB, T2050, only applies to Figure S4). After loading, the modRNA was washed with eluent A (100 mm TEAA in HPLC‐grade H_2_O, pH 7) followed by a linear gradient of up to 20% eluent B (100 mm TEAA in HPLC‐grade H_2_O, 25% (v/v) acetonitrile, pH 7). Elution was performed over 16.2 CVs with a flow rate of 2.8 mL/min using a linear gradient from 20% to 61% eluent B. The final product was subjected to buffer exchange into 1 mM sodium citrate at pH 6.4 using Amicon Ultra‐4 centrifugal filters (10 kDa MWCO) before validating RNA integrity and size on a sodium hypochlorite agarose gel [45] and detecting dsRNA by Dot Blot as described before [46]; with the additions of cross‐linking the RNA to the membrane by UV‐light (3 min at 120,000 µJ/cm^2^) and validating equal loading by methylene blue staining (0.02% methylene blue in 0.3 m sodium acetate).

### 4.9 Formulation and Transfection of modRNA

For transfection of modRNA into cultured cells, RNAiMAX (Thermo Fisher Scientific)‐ modRNA complexes were prepared following the manufacturer's instructions in OptiMEM I medium, with 1 µg of RNA/µl of transfection reagent. 6 µg of modRNA were transfected per one million hiPSC‐CMs. 5 h after adding the transfection mix, it was replaced by full cell culture medium (RPMI1640 and GlutaMAX with HEPES, 2% (w/v) serum‐free B27 supplement). In another approach, lipid nanoparticles (LNPs) were produced by microfluidic mixing of an aqueous RNA solution (modfLuc, modEGFP or modGLA in 50 mm sodium acetate buffer, pH 4.8) and an organic lipid phase (SM‐102 (Cayman Chemical), cholesterol, DSPC, DMG‐PEG2k (Avanti Polar Lipids)—[50:38.5:10:1.5 molar%] 12.5 mm in ethanol) using a NanoAssemblr Ignite system (Cytiva) at a flow rate ratio of 3:1 (aqueous:organic), an N/P of 8 and a total flow rate of 12 mL/min. For buffer exchange and concentration of LNPs ultrafiltration was performed using Amicon Ultra centrifugal filters (MWCO 30 kDa, Merck Millipore), before sterile filtration with 0.2 µm Acrodisc syringe filters (Cytiva). Finally, physicochemical particle properties, including polydispersity index, z‐average diameter, and number mean diameter were determined by dynamic light scattering (Zetasizer Nano ZS; Malvern Panalytical) and using a fluorescence‐based RiboGreen assay (Thermo Fisher Scientific) to determine encapsulation efficiency. For transfection, LNPs were added to the respective culture media supplemented with 1 µg/ml of recombinant human ApoE4 (Peprotech).

### 4.10 Caspase Activity Assay

The relative caspase 3/7 activity was determined using the Caspase‐Glo 3/7 kit (Promega) according to the manufacturer's instructions. In brief, cells were seeded into Geltrex‐coated microplates at 30,000 hiPSC‐CMs /cm^2^ one week before performing the assay. Next, 18 h preceding the assay, a doxorubicin (2.56 µm) stress stimulus, diluted in RPMI1640 and GlutaMAX with HEPES, 2% (w/v) serum‐free B27 supplement, was applied to respective wells. To perform the assay, the caspase‐Glo reagent was prepared and added to lysed cells. Following incubation for 1 h at 37°C, luminescence was recorded with a Synergy HT plate reader (Agilent Technologies).

### 4.11 Mitochondrial Membrane Potential Measurement

The mitochondrial membrane potential was indirectly measured using the fluorescent dye tetramethyl rhodamine ethyl ester (TMRE). 24 h prior to the assay, the respective cells were challenged with 1 µm doxorubicin in RPMI1640 and GlutaMAX with HEPES, 2% (w/v) serum‐free B27 supplement. The hiPSC‐CMs were dissociated into single cells (0.25% Trypsin/EDTA for 5 min at 37°C) and stained adhering to the manufacturer's instructions provided with the TMRE‐Mitochondrial Membrane Potential Assay Kit (Abcam). The cells were strained through a 100 µm strainer and analyzed on a CytoFLEX S (Beckman Coulter) cytometer (Ex/Em: 488/575 nm).

### 4.12 Measurement of Cellular ROS

Cellular ROS was measured via the DCFDA/H2DCFDA Cellular ROS Assay Kit (Abcam) following the manufacturer's instructions. In brief, hiPSC‐CMs were seeded onto GelTrex‐coated microplates at 30 000 cells/cm^2^. At least one week later, staining was performed for 45 min at 37°C with 25 µm 2’,7’‐dichlorofluorescin diacetate (DCFDA). Then, the cells were washed with DPBS before adding 100 µm H_2_O_2_ in assay buffer or buffer only as a background control. Finally, the plates were imaged every 30 min for 6 h total using a Cytation 1 (Agilent Technologies) device (Ex/Em: 485/535 nm). Data were analyzed by subtracting the background from the raw read values and plotting the results to calculate the area under the curve.

### 4.13 Calcium Imaging

Calcium transients of individual Fura2‐AM‐loaded hiPSC‐CMs, attached to MatriGel‐coated 18 mm glass cover slips, were recorded on a dual excitation fluorescence photomultiplier setup (IonOptix). First, hiPSC‐CMs were loaded with Fura2‐AM (1.5 µm Fura2‐AM in RPMI1640 medium containing GlutaMAX supplement with HEPES, 2% (v/v) serum‐free B27 supplement) for 30 min at 37°C and 5% CO_2_. Following two 15‐min washing steps in medium only, cover slips were transferred to a custom‐built perfusion chamber and paced at 0.5 Hz and 25 V (MyoPacer Cell stimulator; IonOptix) under constant flow of CM imaging solution (117 mm NaCl, 20 mm HEPES, 10 mm creatine, 10 mm D‐glucose, 5.7 mm KCl, 5 mm sodium pyruvate, 1.25 mm CaCl_2_, 1.2 mm NaH_2_PO_4_, 0.66 mm MgSO_4_; pH 7.4 at 37°C). Calcium transients of randomly selected hiPSC‐CMs that reacted to pacing, were recorded by detecting emitted fluorescence at 510 nm after excitation at both 340 and 380 nm. Finally, 20 calcium transients were averaged per cell using the IonWizard software (IonOptix, Version 6.5) and the parameters baseline ratio (R), ratio amplitude (R), maximum ratio increase velocity (R/s), time to peak (s), maximum decay velocity (R/s), 50 and 90% decay time (s) and exponential decay constant tau (s) were calculated. Outliers in the data were identified using ROUT (Q = 5%). Caffeine‐induced calcium transients were measured in hiPSC‐CMs that reacted to pacing. Pacing was stopped, before applying caffeine via a secondary perfusion pump into the perfusion chamber. The caffeine‐induced calcium amplitude was defined as the maximal increase in the Fura 2 ratio amplitude (R) relative to the baseline ratio (R) of the respective cell, before application of caffeine.

### 4.14 Western Blot

Cells were lysed in RIPA buffer (+ protease & phosphatase inhibitor) by sonication. The protein concentration in lysates was determined by Pierce BCA protein assay kit (Thermo Fisher Scientific). Next, total protein (25 µg) was separated on an SDS polyacrylamide gel and then transferred onto a polyvinylidene membrane by wet blotting (Bio‐Rad). For phospho‐PKA substrate immunoblots, total protein per lane was assessed by Ponceau S staining. The primary antibodies were used as follows to detect the respective proteins: GAPDH (1:20000, ab8245, Abcam); PLN (1:5000, A010‐14, Badrilla); phospho‐Ser16‐PLN (1:5000, A010‐12AP, Badrilla); SERCA2a (1:1000, MA3‐919, Thermo Fisher Scientific); NCX1 (1:10000, ab177952, Abcam); Phospho‐PKA Substrate (RRXS*/T*, 1:2000, 100G7E, Cell Signaling Technology). Prior to reprobing, membranes were stripped in Restore PLUS Western Blot Stripping Buffer (Thermo Fisher Scientific).

### 4.15 Contractility Measurement of hiPSC‐CMs

Immediately prior to contractility assessment, the culture medium was replaced with fresh cardiomyocyte maintenance medium, and hiPSC‐CMs were allowed to equilibrate at 37°C. Contractile activity was recorded using an Olympus IX83 inverted microscope equipped with a 20× air objective and a metal‐halide illumination source. Image sequences were captured with a cooled CCD camera (Orca‐R2, Hamamatsu Photonics) while electrical field stimulation was applied at 1 Hz using a MyoPacer stimulator (IonOptix). Beating hiPSC‐CMs were imaged for 60 s using CellSens Dimension software (v1.16, Olympus). The recordings were subsequently trimmed to 10‐s segments and analyzed for contractile parameters using the MUSCLEMOTION plugin in ImageJ/FIJI [47] together with a custom‐written R script.

### 4.16 Preparation of Living Myocardial Slices From Porcine Heart Tissue

After sedation with intramuscular 8 mg/kg azaperone (Stresnil, Janssen‐Cilag GmbH, Neuss, Germany)/ 15 mg/kg ketamine 10% and 10 mg atropine 1%, the animals underwent ear vein cannulation, and narcosis was induced (2 mg/kg propofol, propofol 1% MCT, Fresenius Kabi Austria GmbH, Graz, Austria; 10 µg/kg fentanyl, Fentanyl‐Jannsen, Janssen‐Cilag GmbH). Anaesthesia was maintained throughout the whole experiment by mechanical ventilation using a Getinge flow‐c ventilator and 1.5 Vol% isoflurane (Piramal Critical Care GmbH, Hallbergmoos, Germany) via an endotracheal tube (inspired oxygen tidal volume, 10 mL/kg) and fentanyl (10 µg/kg/h), accompanied by Ringer's solution at an infusion rate of 4 mL/kg/h. We adapted the respiratory frequency to preserve physiologic end‐tidal carbon dioxide levels (35–40 mmHg). Body temperature was maintained at 38.2 ± 0.2°C using convective heating (Warm Touch 5300A, Covidien, Dublin, Ireland). Finally, the animal was euthanized for reasons unrelated to present study by intravenous administration of 80 mg/kg sodium pentobarbital. Organ harvesting was approved by the LAVE NRW as a 3R project for the shared use of organs for scientific purposes under reference number AZ 81‐02.04.2022/M2025‐1022.

The heart, including the pericardium, was removed, and moved in ice‐cold Tyrode's solution until further processing. Cardiac tissue was embedded in low‐melting agarose for sectioning into 300‐µm‐thick living myocardial slices (LMSs) with a precision vibratome (Campden Instruments). Slices were maintained in cold Tyrode's solution, randomly assigned to treatment groups, trimmed to ∼6.5 × 6.5 mm, aligned along the myocardial fiber direction, and mounted on plastic scaffolds or force‐measurement rings (InVitroSys). Porcine LMSs were cultured at 37°C in a biomimetic auxotonic culture system with continuous electrical stimulation at 0.2 Hz. Preparation of tissue lysates and subsequent α‐galactosidase enzyme activity assay was performed as described in the enzyme activity method section; with the exception that tissue was lysed for 1 h instead of 30 min. LDH activity was measured in supernatants by the institute of clinical chemistry and central laboratory at MHH.

### 4.17 Preparation and Treatment Of Human Precision‐Cut Liver Slices (hPCLiS)

Human liver material was obtained from a 68‐year‐old male patient diagnosed with cholangiocarcinoma undergoing partial hepatectomy at Hannover Medical School (MHH). The patient prospectively gave consent for the tissue being utilized for performed research (approved by MHH ethics committee and in accordance with relevant guidelines and regulations; application #10855_B0_K_2023, Viszeralchirurgie MHH). Lamellated tissue with a thickness of approximately 1 cm was transferred to the laboratory on ice submerged in 1× Krebs‐Henseleit buffer. As described in previous publications, cylindrical cores of 8 mm in diameter were excised from tissue pieces with an electrical drill and placed in ice‐cold Belzer UW storage solution (Bridge to Life Ltd., London, UK) until preparation of hPCLiS with a thickness of 250 to 300 µm using a Krumdieck tissue slicer MD6000 (Alabama Research and Development, Munford, AL, USA) [48, 49]. To remove any debris from the cutting procedure, hPCLiS were places in 12‐well plates and washed with 1 mL Gibco William's E Medium (WME) supplemented with Gibco GlutaMAX‐I, 25 mm D‐glucose and 50 mg/L gentamicin (all Thermo Fisher Scientific, Waltham, MA, USA) for 1 h at 37°C under constant orbital rocking at 80 rpm and a humidified atmosphere containing 80% O2 and 5% CO2. For further cultivation, medium was exchanged to WME containing supplements as described before as well as insulin‐transferrin‐selenium (ITS‐solution II, PAN‐Biotech GmbH, Aidenbach, Germany). After a further 24 h, medium was collected and stored at −80°C and hPCLiS were treated with LNP formulations at different concentrations or respective sucrose buffer control. Medium was exchanged every 24 h. hPCLiS were snap‐frozen and stored at −80°C until further processing. Harvested supernatants were immediately transferred to −80°C until subsequent analyses were performed. AST and ALT activity was measured in supernatants by the institute of clinical chemistry and central laboratory at MHH. Tissue lysates were prepared, and α‐galactosidase enzyme activity assay was performed as described for porcine LMS.

### 4.18 WST‐1 Cell Viability Assay

The influence of modRNA transfection on cell viability of hiPSC‐CMs was assessed by WST‐1 assay (Roche, #5015944001) according to the manufacturer's recommendations.

### 4.19 Profiling of Glycosphingolipid‐Derived Glycans

Analysis of glycans derived from glycosphingolipids was performed by multiplexed capillary gel electrophoresis coupled to laser‐induced fluorescence detection (xCGE‐LIF) as described previously [50]. In brief, glycans were cleaved off glycosphingolipids using ceramide glycanase. The resulting free reducing ends were labelled with the fluorescent dye 8‐aminopyrene‐1,3,6‐trisulfonic acid (APTS), which enabled glycan detection during the subsequent capillary gel electrophoresis. Glycan subtypes were identified by comparison of migration times to a migration time database containing the respective glycans.

### 4.20 Ethics Statement

All procedures involving human material were approved by the local ethics committee of Hannover Medical School (approval nos. 409 and 3668‐2017 for generation and use of hiPSC; and #10855_B0_K_2023, by the Viszeralchirurgie MHH for PCLiS experiments). HiPSCs and hPCLiS in this study were obtained and used in accordance with institutional and national ethical guidelines. Donors provided informed consent for the generation and research use of hiPSC lines, and hepatic tissue being utilized for research. The research involving porcine tissue was approved by the Landesamt für Verbraucherschutz und Ernährung Nordrhein‐Westfalen (LAVE), Germany. The approval was granted under the reference number AZ 81‐02.04.2022/M2025‐1022 (initial application). Ethical approval from the relevant national authority was obtained prior to the commencement of the study.

### 4.21 Statistics

Statistical analyses of data were performed using GraphPad Prism (v9.1.0). Data are presented as mean (SD). To compare means between two groups, an unpaired two‐tailed Student's *t*‐test was used. For comparisons among three or more groups, ordinary one‐way ANOVA was followed by Tukey's multiple comparisons test when homogeneity of variances could be assumed. When variances were unequal, Brown‐Forsythe and Welch's ANOVA were used and followed by Dunnett's T3 multiple comparisons test. For experiments involving two independent variables, two‐way ANOVA followed by Tukey's multiple comparisons test was applied. Post–hoc *p*‐values are only reported when the corresponding ANOVA indicated a statistically significant difference (*p* ≤ 0.05).

## Author Contributions

MJ designed experiments, analyzed data, and drafted the manuscript. MJ, JHo, TT and CB conceptualized the study, and revised the manuscript. MJ, LO, SE, JLY contributed to the production of modRNA and LNPs. EM and MJ performed contractility assessment. NW provided significant support with calcium imaging. NW, IK, LE, WA, and JL performed LMS experiments. KS, LF and RP performed PCLiS experiments. CJ, RE and TC planned and performed the prime editing‐based gene correction of the patient‐derived iPSC line. JHe performed electron microscopic analyses. MF and KX performed bioinformatic analysis of transcriptomics data. JB and FB performed analysis of glycosphingolipids. All authors reviewed and approved the final version of the manuscript.

## Funding

Support was received from the German Research Foundation (to JH: DFG grant no. HO 6855/1‐1; to CB and TT: SFB TRR267). In addition, this work was supported by the Fraunhofer lighthouse project “RNAuto” (to CB).

## Conflicts of Interest

TT is the founder and Chief Scientific/Medical Officer of Cardior Pharmaceuticals GmbH, which operates as a fully owned subsidiary of Novo Nordisk A/S Europe (not related to this publication). TT and CB are listed inventors on patents concerning the therapeutic application of RNAs, which have been licensed (unrelated to the work presented here). All other authors report no competing interests.

## Supporting information




**Supporting File**: advs77151‐sup‐0001‐SuppMat.pdf.

## Data Availability

The data supporting the findings of this study will be made available from the corresponding author upon reasonable request.
