# Supplementary material for: Nucleoside‐Modified mRNA Encoding Alpha‐Galactosidase A Ameliorates Fabry Disease Phenotypes in Human IPSC‐Derived Cardiomyocytes
Source: Adv Sci (Weinh). 2026 Aug 13:e77151. Online ahead of print. doi: 10.1002/advs.77151 (PMC13470294; doi:10.1002/advs.77151)
Supplement: Supplementary file 1 [file ADVS-9999-e77151-s001.pdf]

Supporting information

Nucleoside-Modified mRNA Encoding Alpha-Galactosidase A Ameliorates Fabry Disease Phenotypes in Human IPSC-Derived Cardiomyocytes

Malte Juchem[1,2], Sedef Ersoy[1], Lea Oehlsen[1,2], Jia Li Ye[1,3], Natalie Weber[1,4], Elisa Mohr[1,2], Wilson Agyapong[1], Junqing Liu[1], Maximilian Fuchs[1], Ke Xiao[1], Christopher Jahn[1], Reto Eggenschwiler[5], Tobias Cantz[5], Julia Beimdiek[6], Falk F. R. Buettner[6], Theresia Kraft[7], Jan Hegermann[8], Ivonne J. Knorr[9], Lisa Ernst[9], Linda Feldbrügge[10], Resa Puffert[10], Kevin Schmidt[2,11], Christian Bär[1,2,3,11#], Jeannine Hoepfner[1#], Thomas Thum[1,3*#]

# equal contribution

* Corresponding Author

This file includes:

Figures S1-S14

Uncropped images of immuno blots

Nucleotide sequences of plasmids and transcribed modRNA

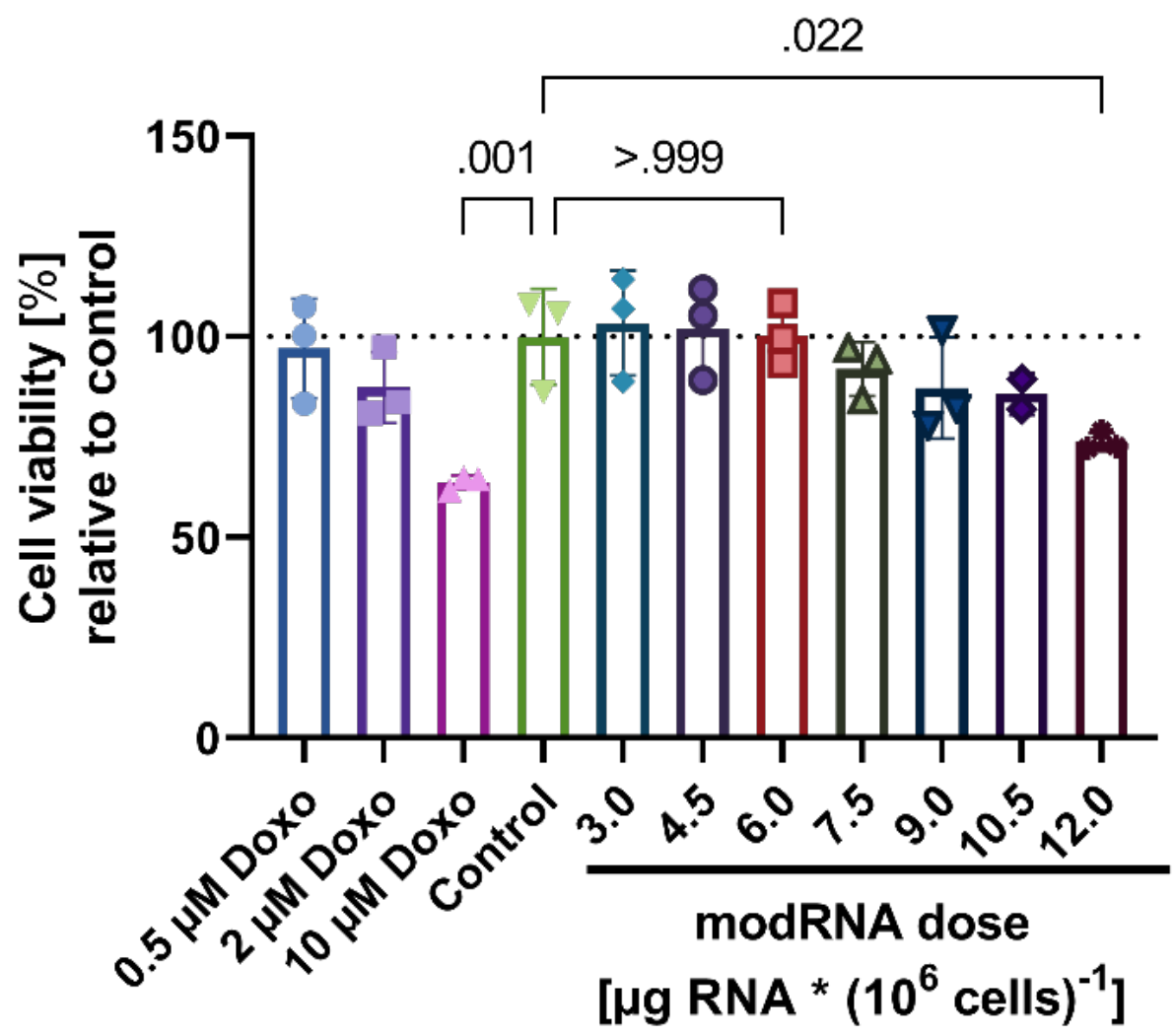

Figure S1 – Cell viability of hiPSC-CMs in response to escalating doses of modGLA

Cell viability of hiPSC-CMs as assessed by WST-1 assay 24 hours after transfection with increasing doses of modGLA or after doxorubicin treatment, which served as a positive control for cardiotoxic effects. The cell viability begins to drop off with modGLA doses of 7.5 µg RNA * (10^6 cells)$^{-1}$. Statistical analysis was performed using one-way ANOVA followed by Dunnett's multiple comparisons test, comparing the mean of each group with the control (mean (SD); n = 3 individual wells; α = 0.05).

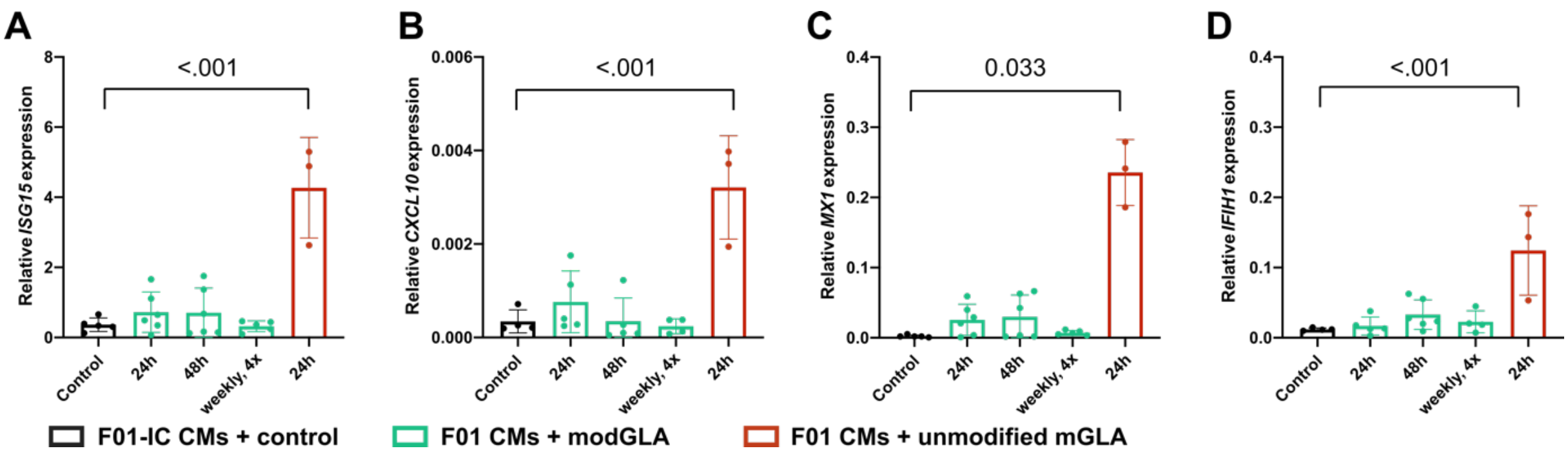

Figure S2 – Reduced innate immune response of hiPSC-CMs to modified compared to unmodified GLA mRNA

Innate immune response of hiPSC-CMs against in vitro transcribed nucleoside-modified (modGLA) or unmodified (mGLA) as assessed by RT-qPCR, measuring the expression of (A) *ISG15*, (B) *CXCL10*, (C) *MX1*, (D) *IFIH1*; 24, 48 hours, and 1 week following the last dose of 4 weekly doses (6 µg RNA * (10^6 cells)$^{-1}$). Unmodified mGLA, was included as a positive control for immunogenicity. Statistical analysis was performed using ANOVA followed by Dunnett's multiple comparisons test, comparing the mean of each group with the control (mean (SD); n = 3 individual wells; α = 0.05).

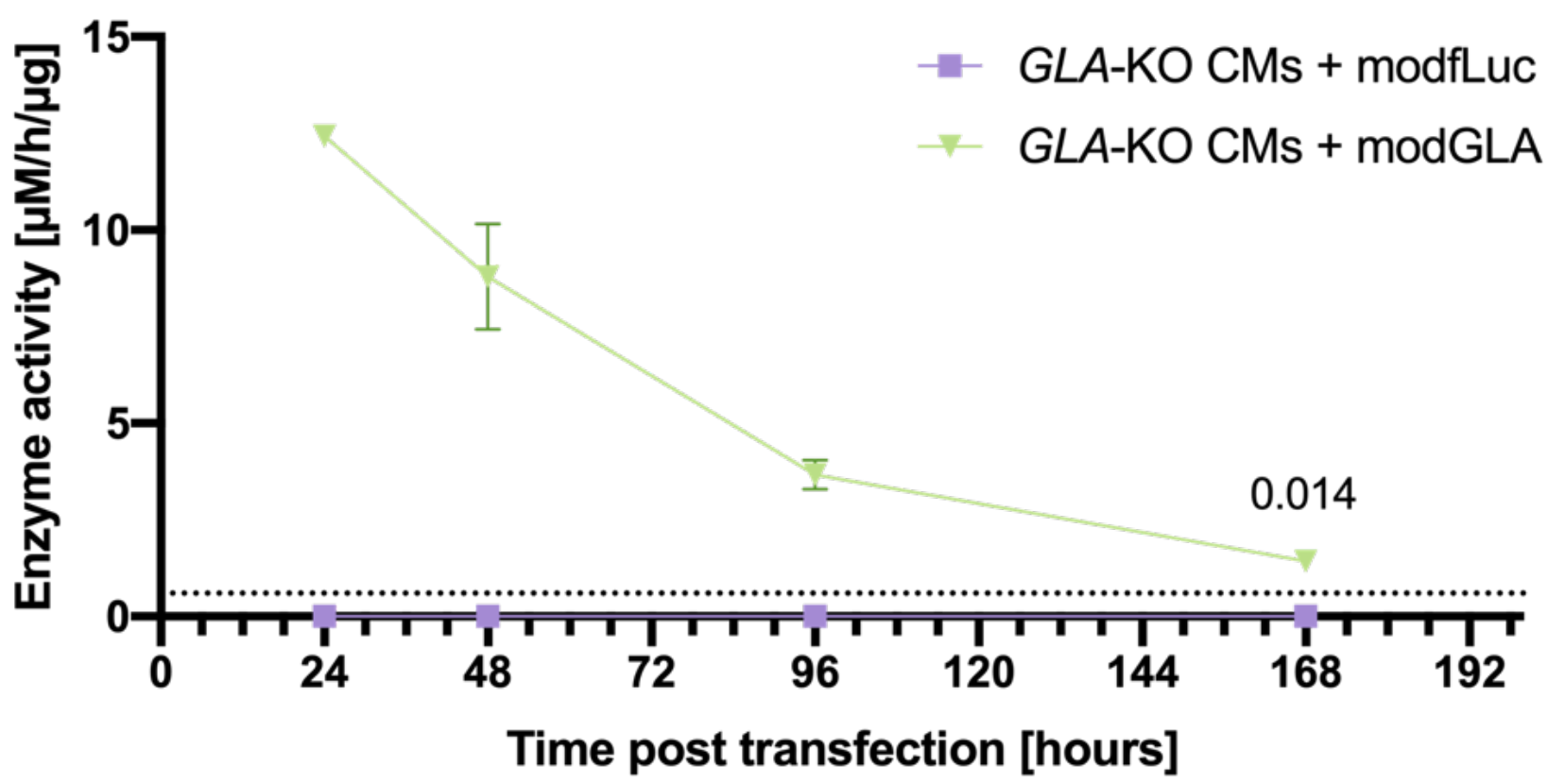

Figure S3 – Single dose enzyme activity kinetics

HiPSC-CMs were transfected with a single dose of therapeutic modGLA or modfLuc control RNA. The α-Gal A enzyme activity was analyzed by enzyme activity assay, from 24 hours to 168 hours following transfection (n = 3 independent differentiations; dotted line represents mean α-Gal A enzyme activity in WT CM; Kruskal-Wallis test vs. WT and Dunn's correction for multiple testing).

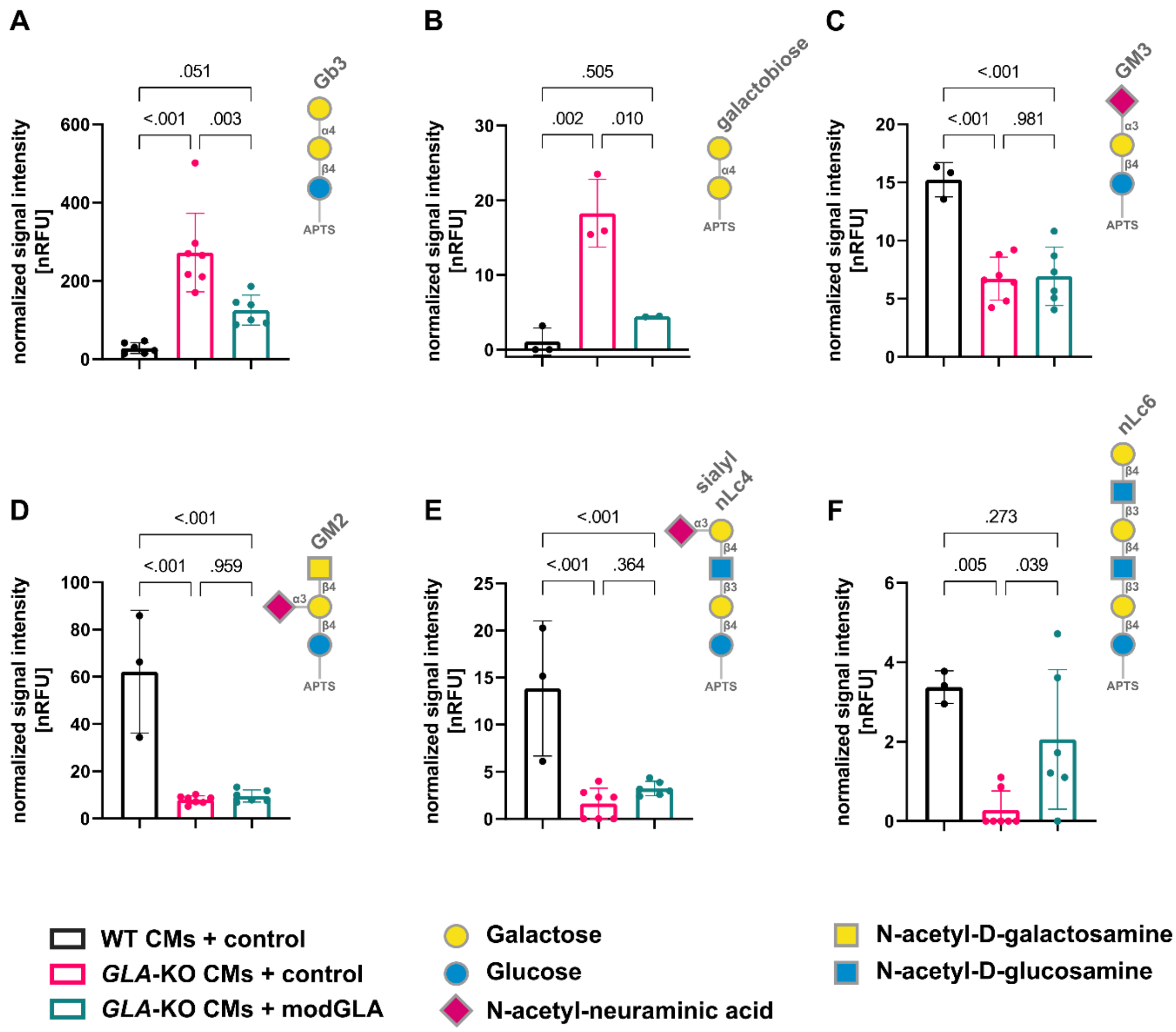

Figure S4 - xCGE-LIF-based glycan profiling reveals altered glycosphingolipid composition in *GLA*-KO CMs

Quantification of glycosphingolipid-derived glycans was conducted in 65-days-old WT and *GLA*-KO CMs (modEGFP control treated) and modGLA-treated *GLA*-KO CMs to assess the therapeutic effect of a single dose of modGLA; one week following transfection. Assigned glycans are presented as pictograms. A single dose of modGLA significantly normalized Gb3, galactobiose, and nLc6 glycan levels. (n = 2-7 independent differentiations, mean (SD), ordinary one-way ANOVA followed by Tukey's multiple comparisons test; α = 0.05).

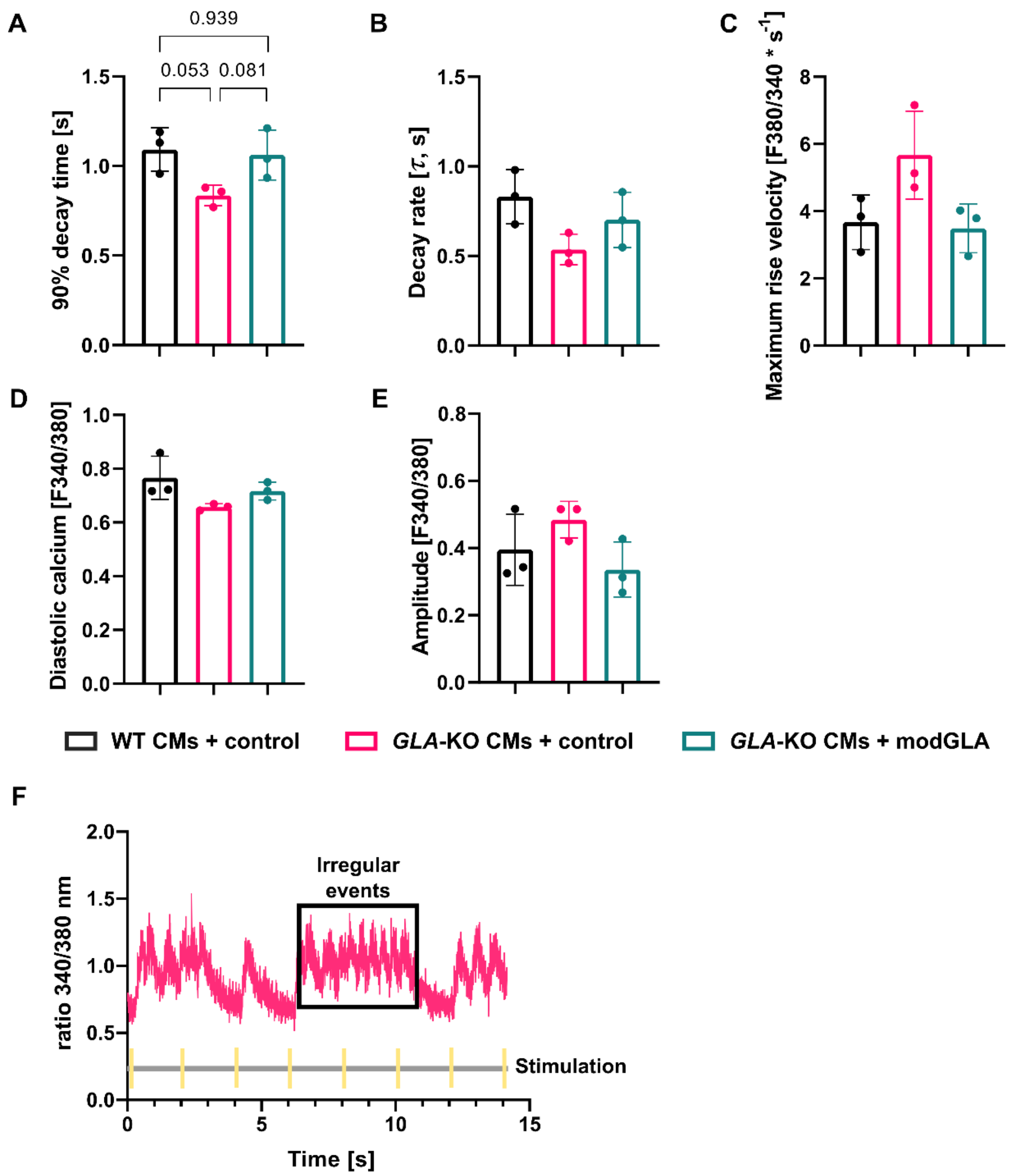

Figure S5 – Potential signs of altered calcium handling in *GLA*-KO CMs

Fura 2-based calcium imaging was used to determine the (A) 90% decay time of calcium transients [s], (B) the decay rate τ [s], (C) the maximum rise velocity as determined by the change in the ratio metric Fura 2 fluorescence per second [F340/380 s^{-1}], (D) the diastolic calcium levels and (E) the transient amplitude [F340/380], (n = 3 independent differentiations, control = modfluc, calcium transients of 20-39 cells were recorded per replicate and group, mean (SD), ordinary one-way ANOVA followed by Tukey's multiple comparisons; α = 0.05. Test performed on log-transformed data). (F) Exemplary depiction of arrhythmic calcium transients detected in a *GLA*-KO CM (highlighted by black box labelled "irregular events"). In contrast, a regular transient precedes the black box between electrical stimuli at 4 and 6 seconds.

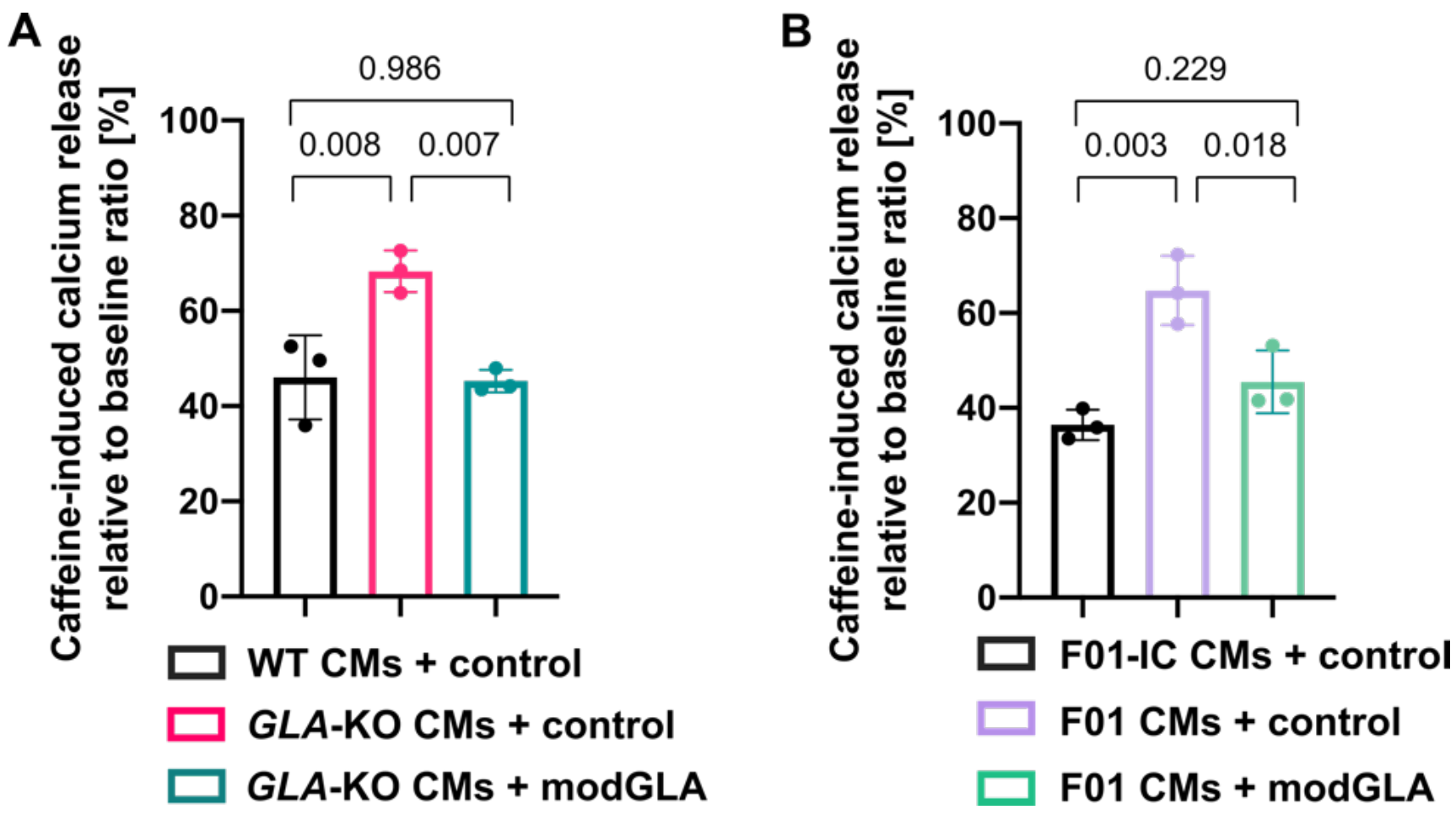

Figure S6 – A-galactosidase A deficient hiPSC-CMs display increased caffeine-induced calcium transient amplitudes.

Fura-2-based calcium imaging was used to quantify caffeine-induced calcium transients by measuring changes in the ratiometric fluorescence signal [F340/F380] over time relative to baseline in (A) patient-independent and (B) patient-derived Fabry disease cardiomyocytes. Calcium transient amplitude was defined as the maximal change in the Fura-2 ratio following caffeine stimulation (n = 4 independent differentiations per condition; control = modfluc, statistical analysis was performed by One-way ANOVA followed by Tukey's multiple comparisons test, α = 0.05). Caffeine was delivered via a secondary perfusion pump into the recording chamber to ensure rapid and controlled application.

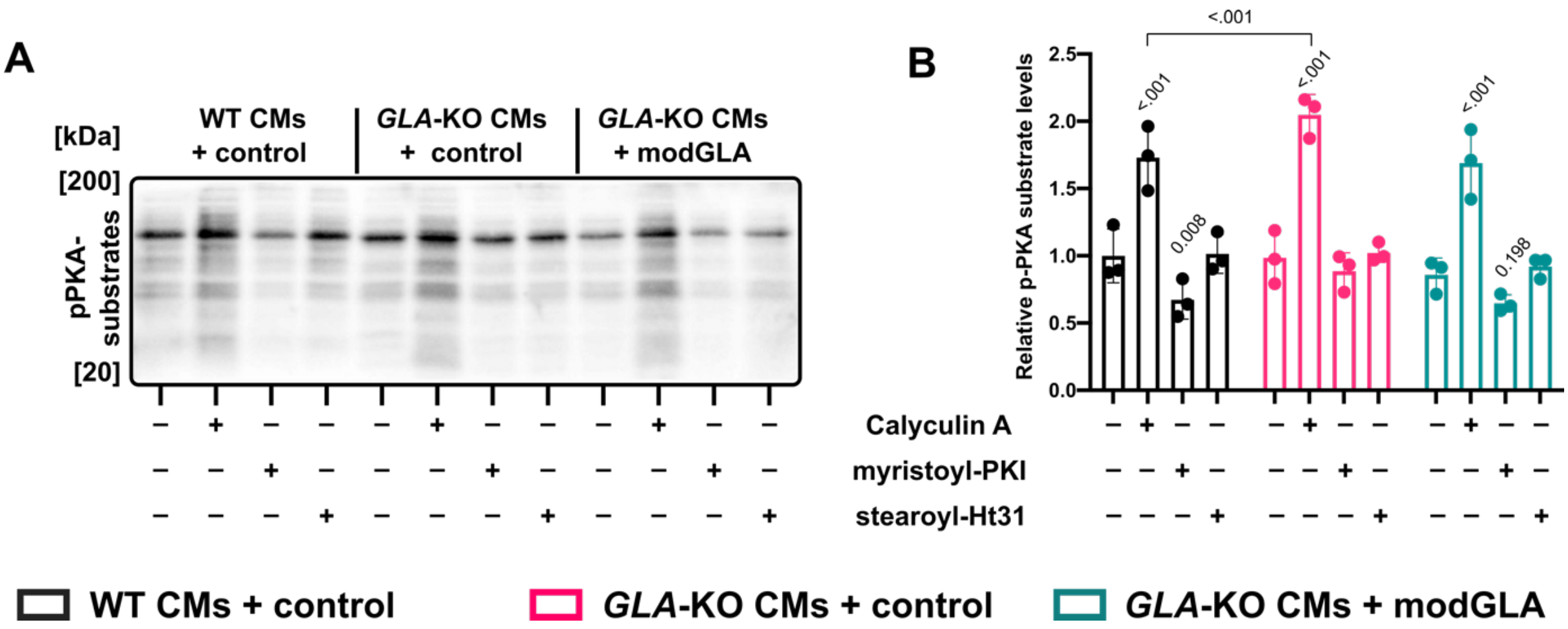

Figure S7 – *GLA*-KO CMs display reduced responsiveness to acute PKA inhibition.

(A) Levels of phosphorylated protein Kinase A-substrates (pPKA substrates) analyzed by Western blot under basal conditions, protein phosphatase inhibition (Calyculin A), PKA inhibition (myristoyl-PKI), and disruption of compartmentation of PKA signaling (stearoyl-Ht31) by disrupting A-kinase anchoring protein (AKAP) and PKA interactions. (B) Densitometric quantification of pPKA-substrate levels normalized to total protein amount per lane determined by Ponceaus S staining (n = 3 independent differentiations, control = modfluc, mean (SD), ANOVA followed by Dunnett's multiple comparisons test; α = 0.05).

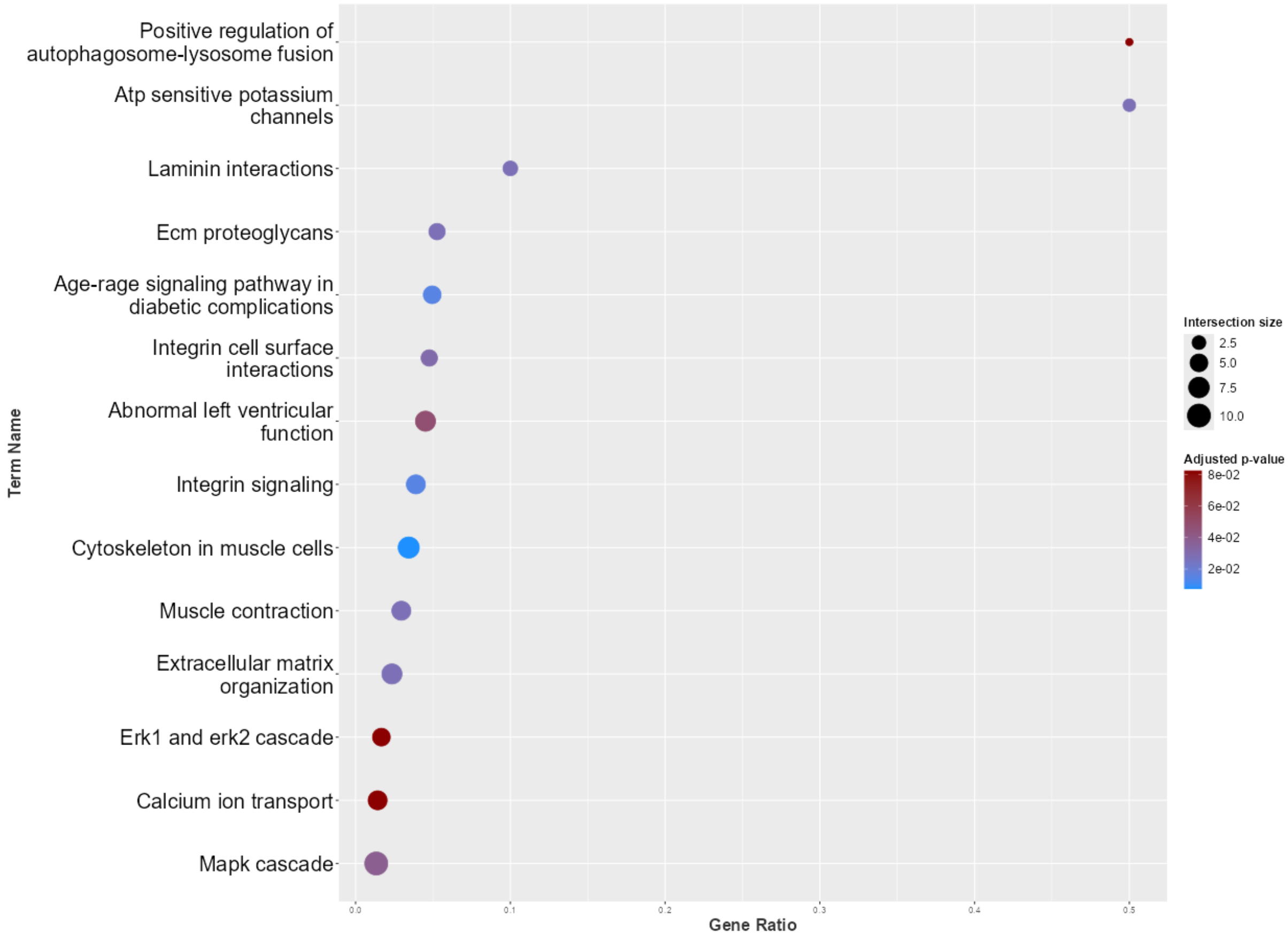

Figure S8 – Pathway enrichment of reciprocally dysregulated genes shared between patient-independent and patient-derived Fabry disease models.

Pathway enrichment analysis illustrated by Bubble plot, based on overlapping DEGs that were significantly dysregulated in both models (between *GLA*-KO cardiomyocytes and WT controls, and between F01 patient-derived cardiomyocytes and F01-IC isogenic controls; baseMean > 5, q < 0.1) but exhibited opposite (reciprocal) regulation between conditions. Only pathways reaching an FDR-adjusted q value < 0.1 are displayed. Bubble size indicates the intersection size, defined as the number of genes from the DEG set contributing to each pathway, while bubble color encodes the adjusted p value.

A- Fabry-distinctive gene pathway networks in patient-independent *GLA*-KO CMs

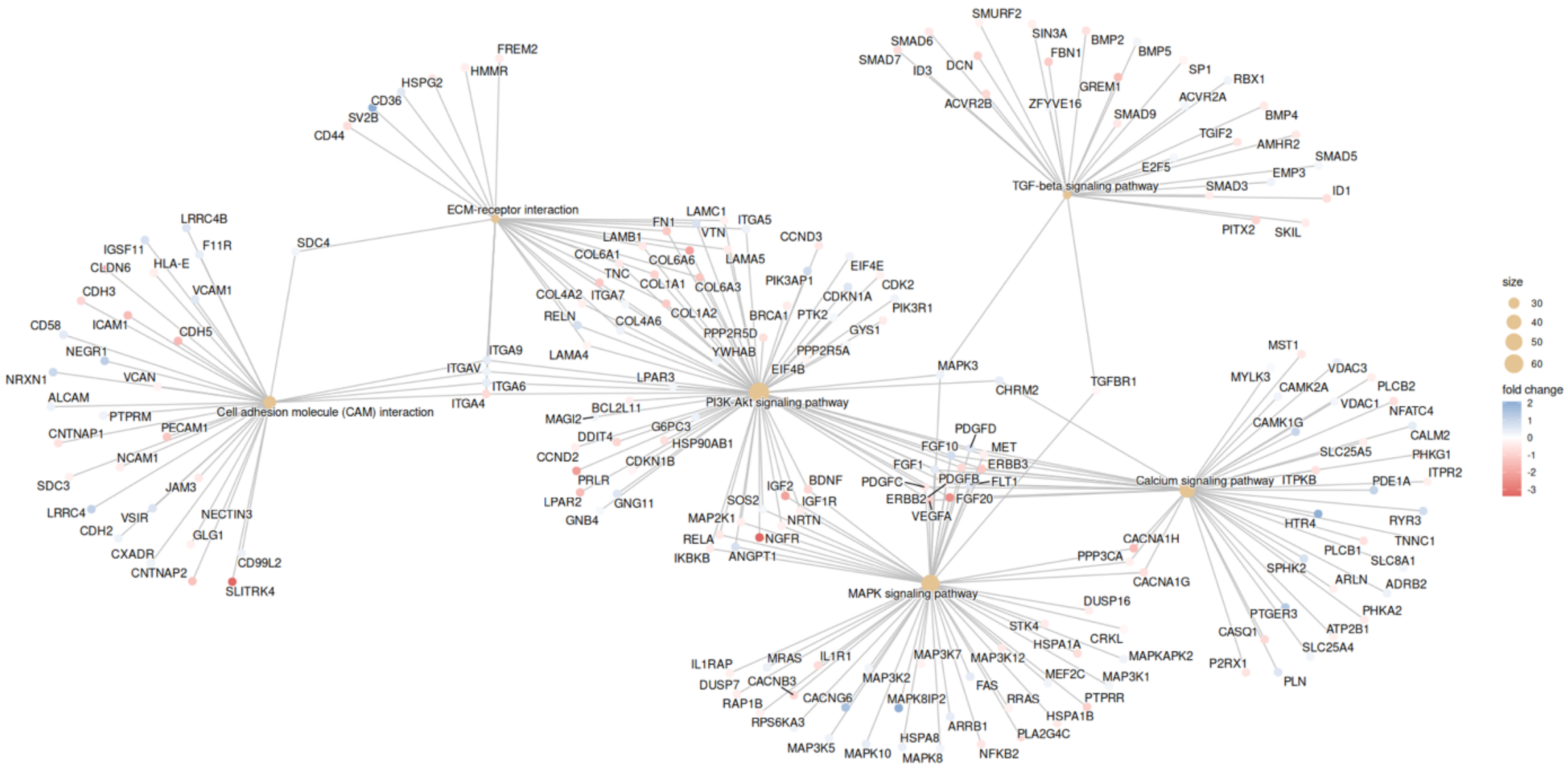

B - Fabry-distinctive gene pathway networks in patient-derived F01 CMs

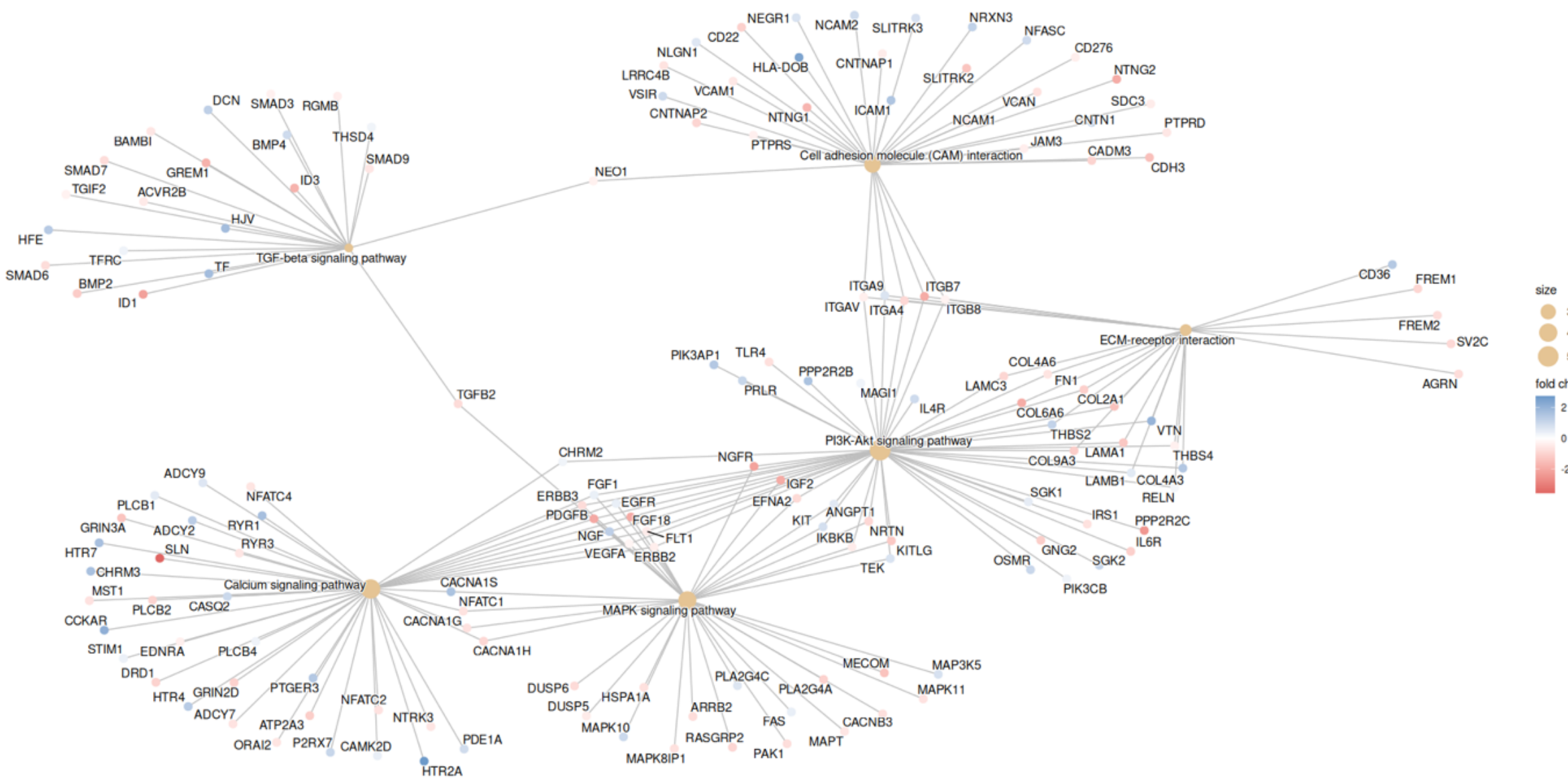

Figure S9 – Fabry-distinctive gene-pathway networks concordantly dysregulated between patient-independent and patient-derived models.

Cnetplots display enriched pathways identified from the set of overlapping DEGs that were significantly dysregulated in both models (baseMean > 5, q < 0.1) with concordant directionality of change. Node colors represent log_2-fold changes derived from RNA-seq comparisons of each disease model versus its respective control (A) *GLA*-KO CMs vs. WT CMs; (B) F01 CMs vs F01-IC CMs.

A - Model-specific gene pathway networks in patient-independent *GLA*-KO CMs

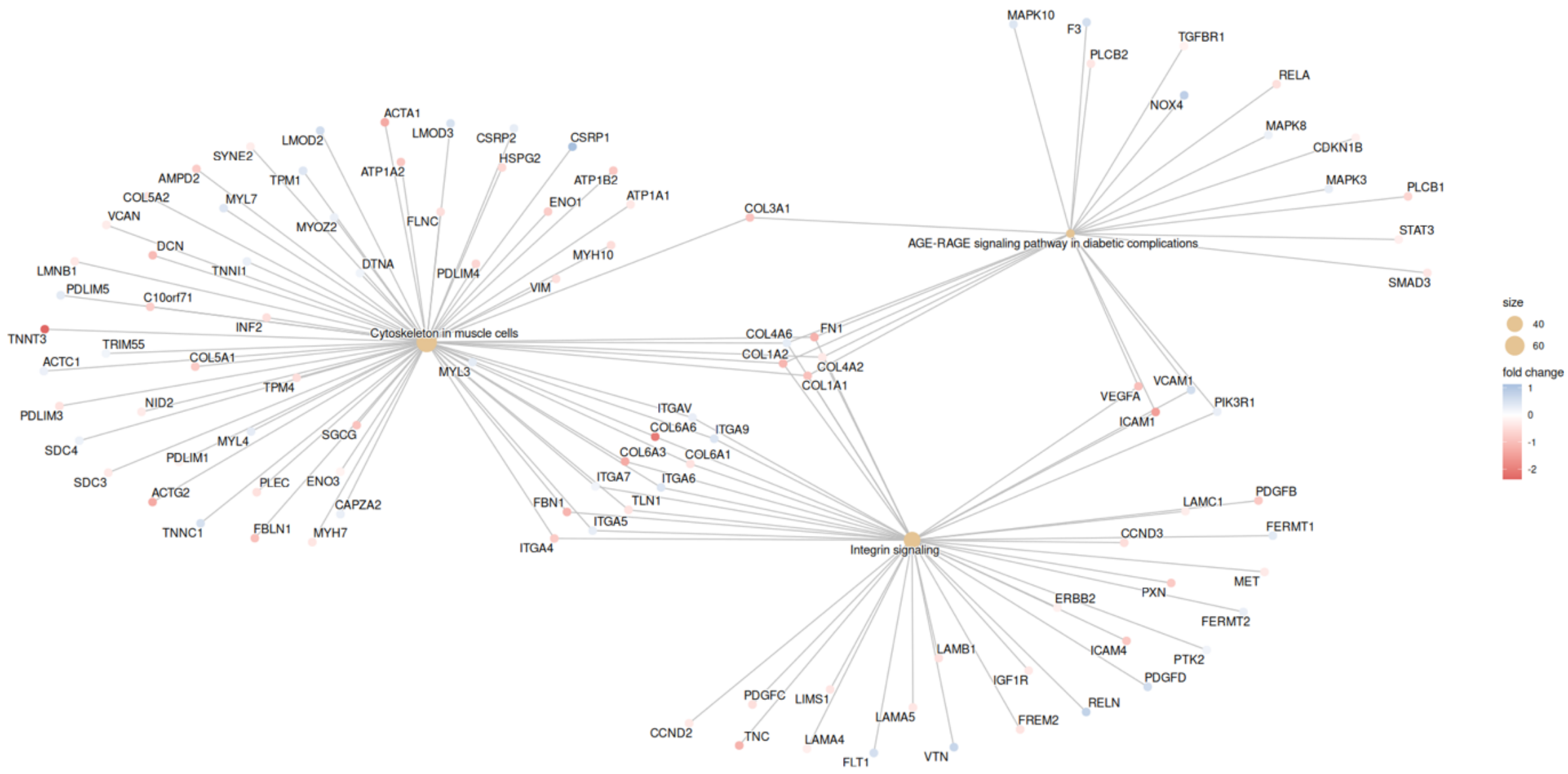

B - Model-specific gene pathway networks in patient-derived F01 CMs

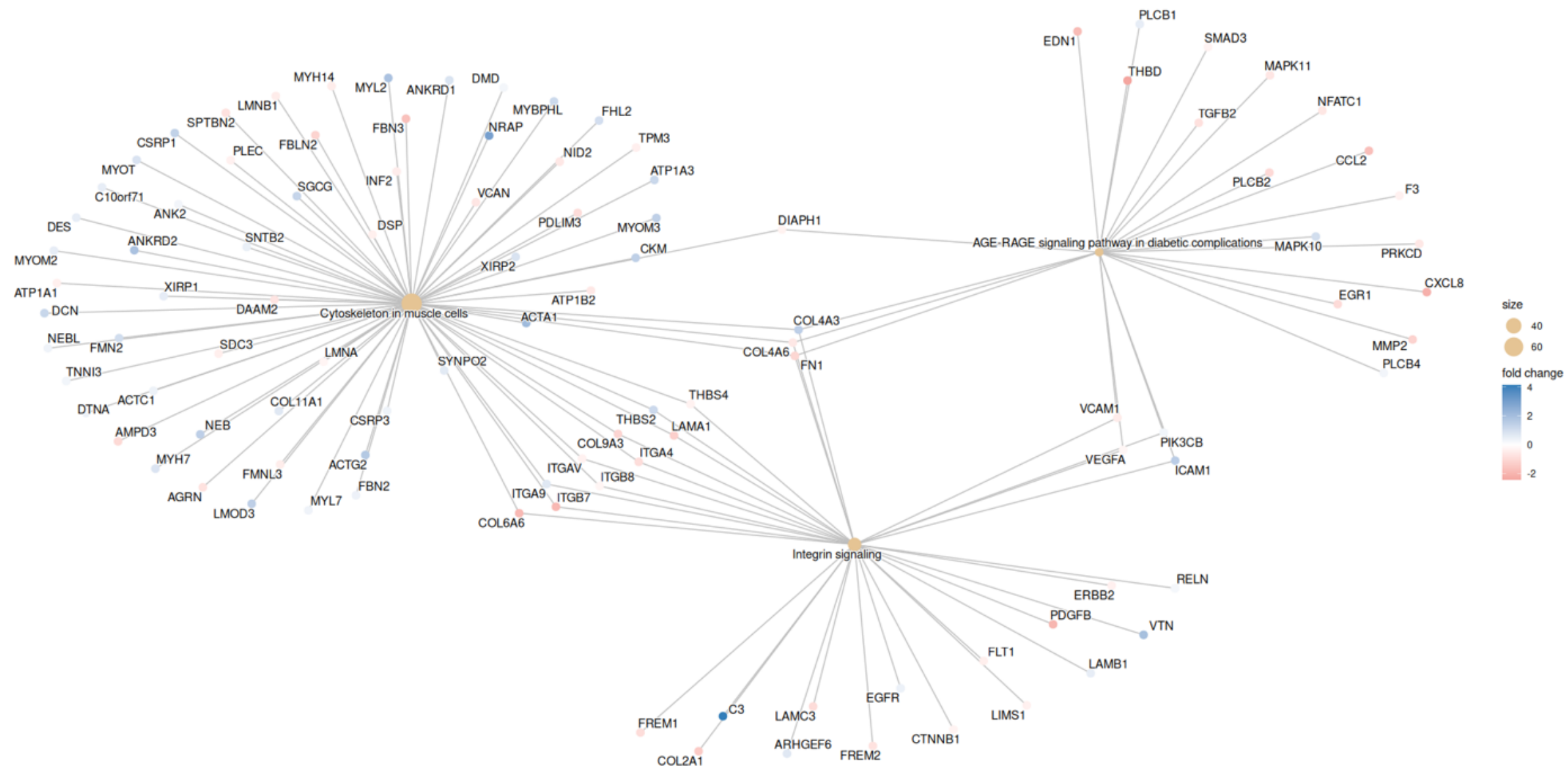

Figure S10 – Model-specific gene-pathway networks reciprocally regulated between patient-independent and patient-derived Fabry disease models

Cnetplots show enriched pathways identified from overlapping DEGs that were significantly dysregulated in both models (baseMean > 5, q < 0.1) but displayed reciprocal directionality of change (i.e., upregulated in one model and downregulated in the other). Node colors represent $\log_2$-fold changes derived from transcriptomic comparisons of each disease model versus its respective control (A) *GLA*-KO CMs vs. WT CMs; (B) F01 CMs vs. F01-IC CMs.

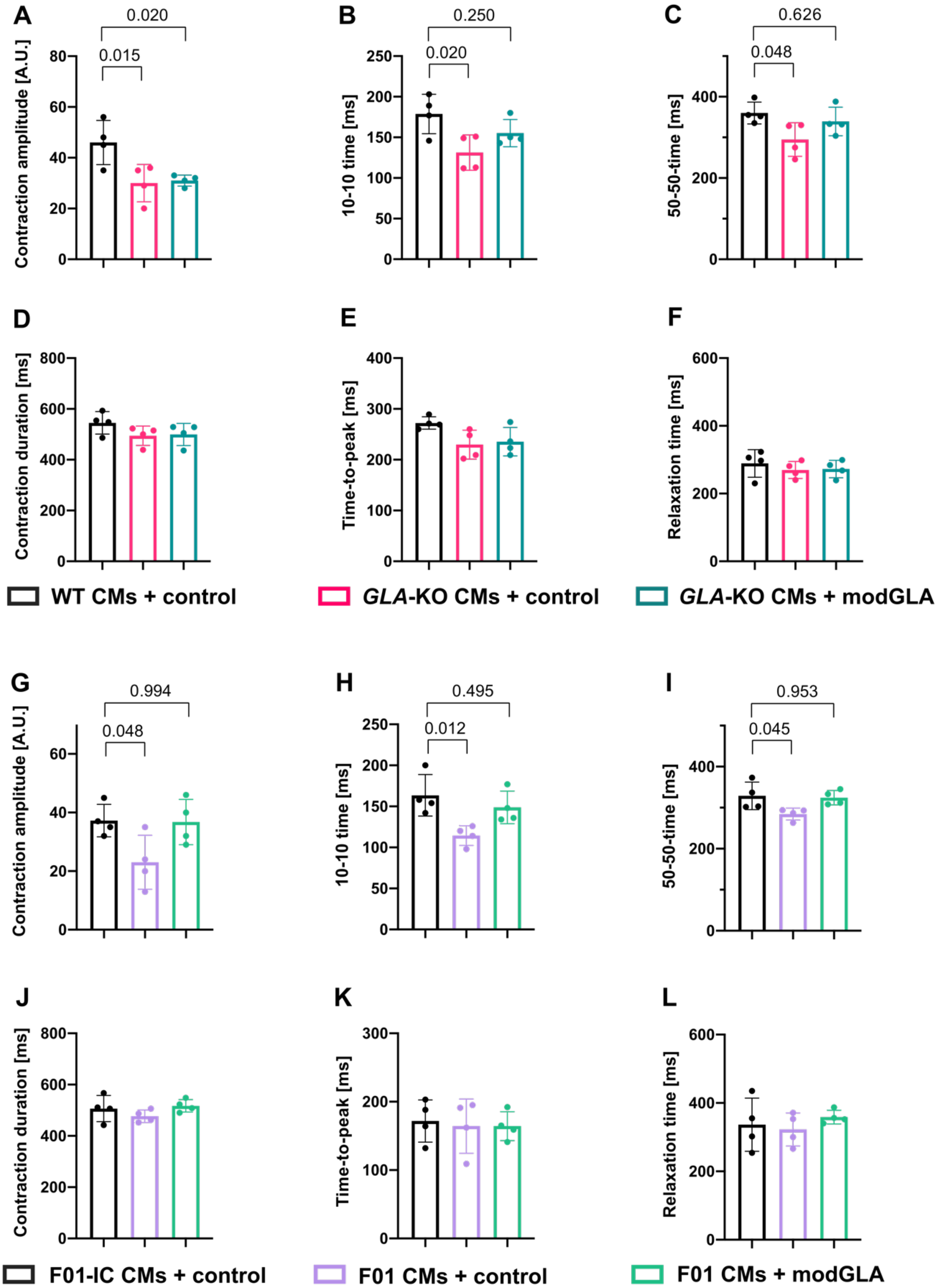

Figure S11 – Altered contractile behavior in α-galactosidase A deficient hiPSC-CMs.

Video-based analysis of hiPSC-CMs' contractility to determine (A, G) contraction amplitude [Arbitrary units (A.U.)], (B, H) 10-10-time [ms], (C, I) 50-50-time [ms], (D, J) contraction duration [ms], (E, K) time-to-peak [ms], (F, L) relaxation time [ms] (n = 4 independent differentiations, control = modfluc, mean (SD), one-way ANOVA followed by Dunnett's multiple comparisons test; α = 0.05).

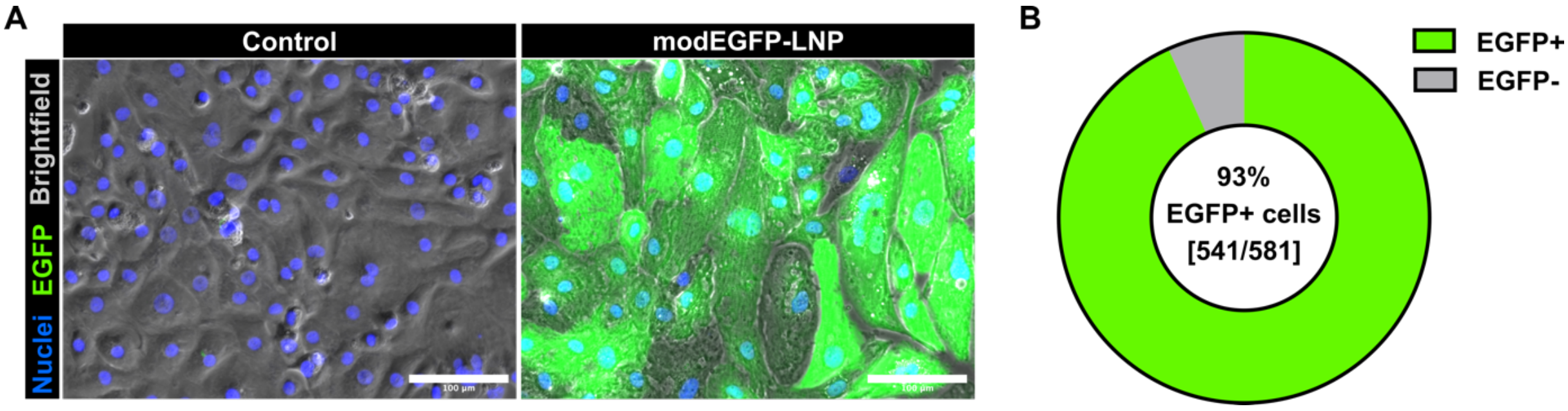

Figure S12 – Lipid nanoparticles efficiently deliver modRNA into hiPSC-derived cardiomyocytes

(A) HiPSC-derived CMs were transfected with either lipid nanoparticles (LNPs) containing modEGFP RNA as a payload (modEGFP-LNP) or an ApoE4-supplemented cell culture medium vehicle control (Control). EGFP expression as analyzed by fluorescence microscopy 24 hours following transfection (40x magnification, scale bar = 100 µm). (B) The fraction of successfully transfected (EGFP+) hiPSC-CMs as determined by cell counting using the *cell counter* ImageJ plugin (n = 581 cells analyzed in total from 12 independent wells; 31-68 cells per well).

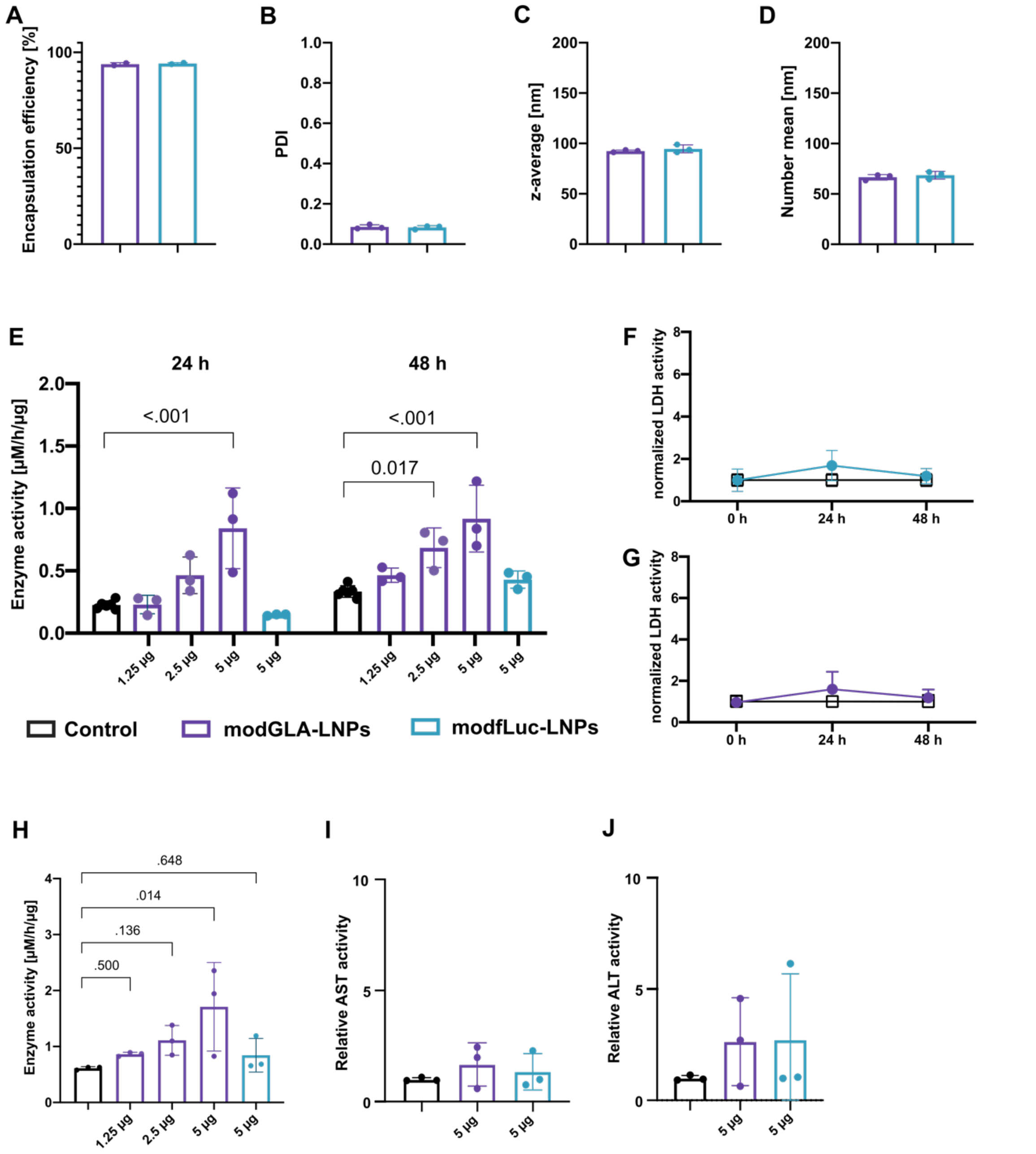

Figure S13 – modGLA lipid nanoparticles increase α-Gal A activity in two human-relevant preclinical models.

Lipid nanoparticles (LNPs) were produced by microfluidic mixing, encapsulating therapeutic (modGLA) or control (modfLuc) RNA. Physicochemical particle properties, including (A) encapsulation efficiency [%] determined by fluorescence-based RiboGreen Assay, as well as (B) polydispersity index (PDI), (C) z-average diameter [nm], and (D) number mean diameter [nm] measured by dynamic light scattering, indicate production of highly homogenous nanoscale LNPs with high encapsulation efficiency for both modRNAs. (E) Transfection of modGLA- and modfLuc-control LNPs into living myocardial slices (LMS; n = 3 LMS per dose and n = 6 LMS per control and time-point; N = 2 pigs), prepared from porcine ventricular tissue, dose-dependently increased α-Gal A activity at both 24 and 48 h following treatment. No significant effect on LDH activity in culture supernatant was detected after transfection with (F) modfLuc-control or (G) modGLA-LNPs. (H) Transfection of human precision-cut liver slices (hPCLiS) with therapeutic or control-RNA-LNPs confirmed the ability of modGLA-LNPs to increase α-Gal A activity 24 h post treatment. Assessment of AST (I) and ALT (J) activity in hPCLiS culture supernatants relative to pre-treatment levels. Statistical analysis was performed by ANOVA followed by Dunnett's multiple comparisons test, comparing the treatment groups with the control group (mean (SD); n = 3 PCLiS; N = 1 donor; α = 0.05).

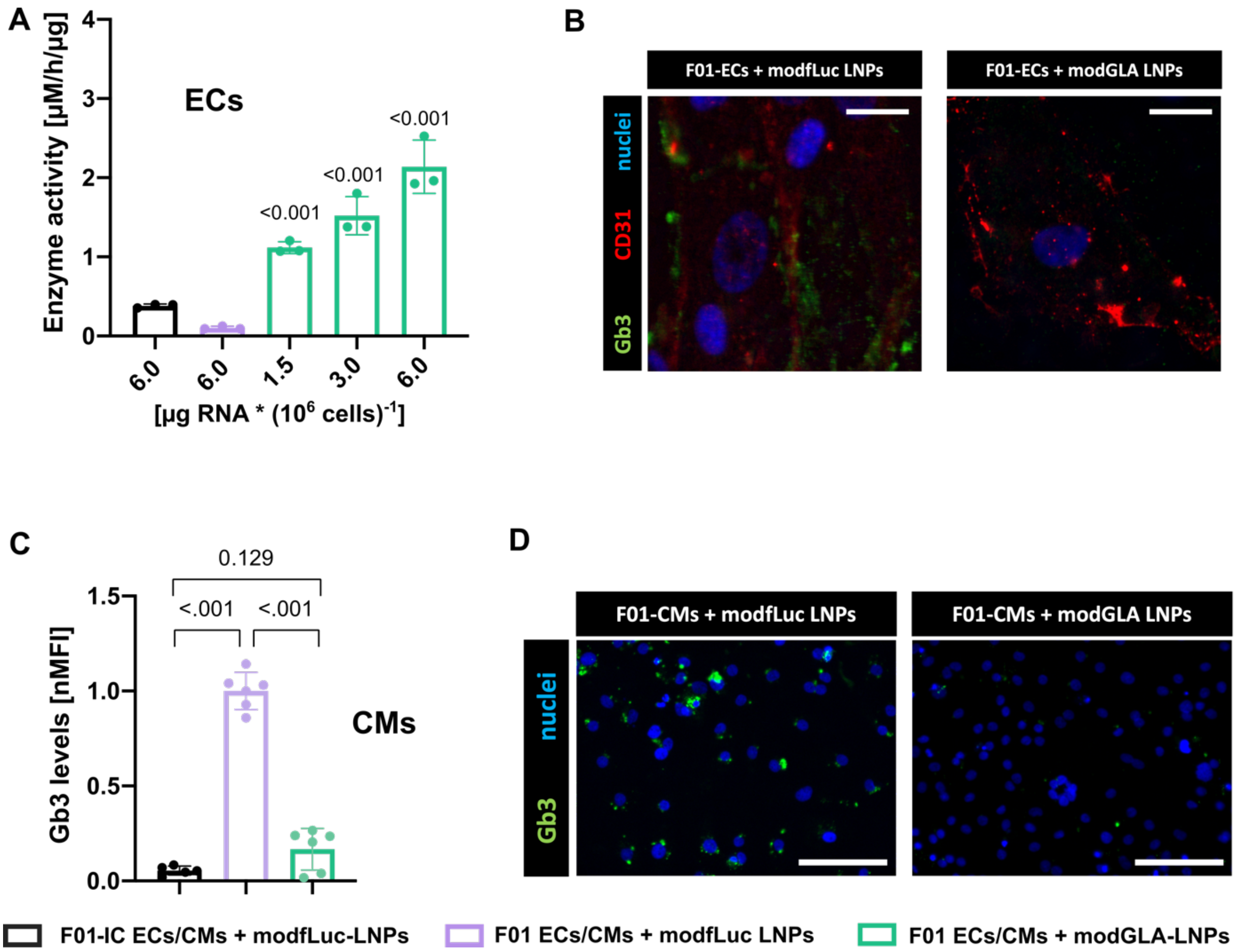

Figure S14 – modGLA lipid nanoparticles rescue primary disease phenotype in Fabry endothelial cells and cardiomyocytes

Lipid nanoparticles (LNPs) encapsulating modGLA or control modfLuc RNA were transfected into (A) Fabry patient-derived hiPSC endothelial cells rescuing α-Gal A enzyme activity. (B) Restored enzyme activity was accompanied by reduction of Gb3 levels as determined by immunocytochemistry and fluorescence microscopy (40x magnification, scale bar = 10 µm). (C) Gb3 deposition in Fabry patient-derived hiPSC-CMs was significantly reduced by modGLA-LNP treatment as analyzed by automated image analysis of (D) immunofluorescence images (20x magnification, scale bar = 100 µm, Brown-Forsythe Welch ANOVA, followed by Dunnett's multiple comparisons test, n = 5 independent differentiations).

Uncropped images of immuno blots

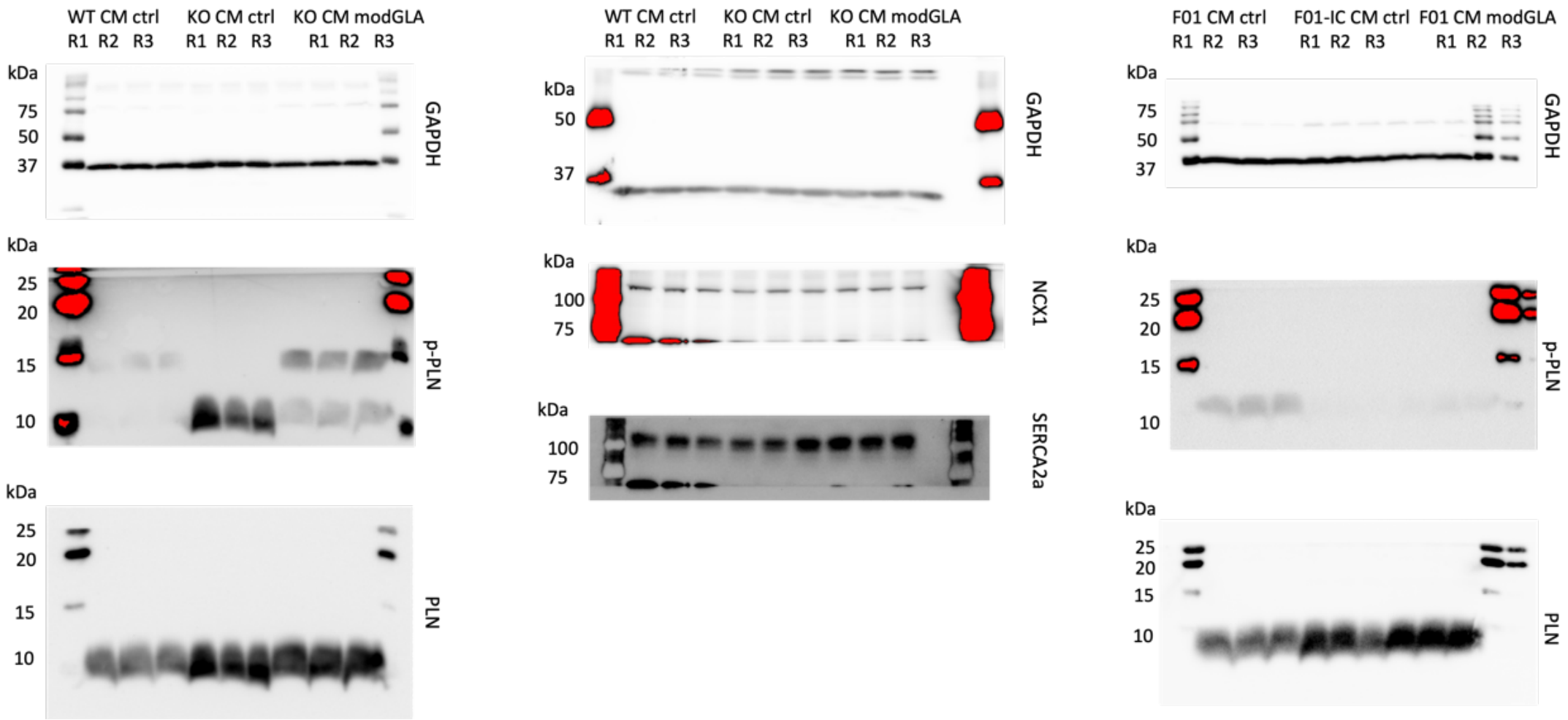

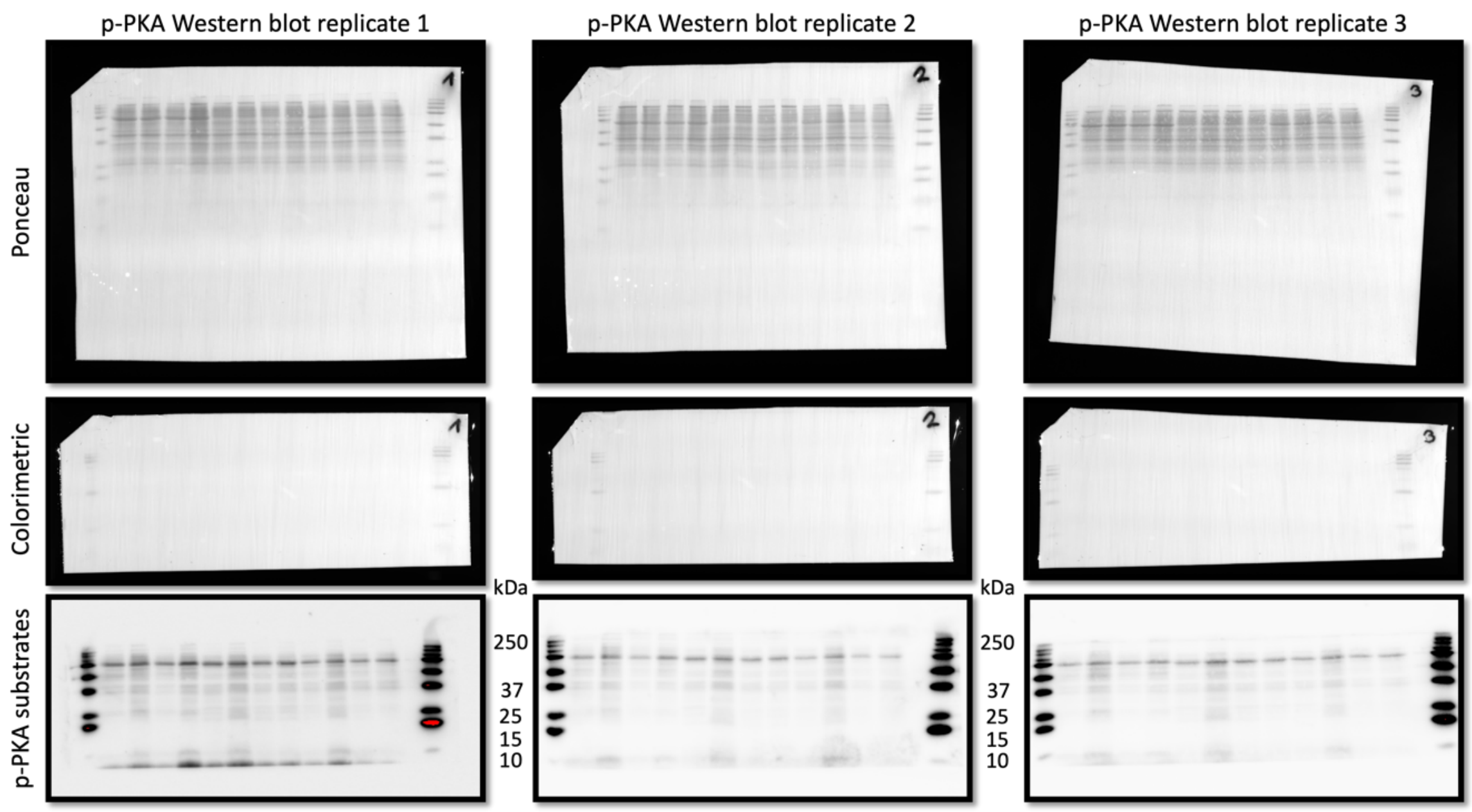

Nucleotide sequences of plasmids and transcribed modRNA

U = N1-methyl-pseudouridine
Cap1 = (7-methyl-guanosine)-triphosphate linkage-(2-*O*-methyladenosine)

>pMIVT.GLA.BbsI

CTAAATTGTAAGCGTTAATATTTTGTTAAAATTCGGATGTGCTGCAAGGCGAGCGGCCGCAGGCCGTCAAGGCCGC
ATGGTACCTGACAGCTGTAATACGACTCACTATAAGGAGAACTCTTCTGGTCCCCACAGACTCAGAGAGAACCCACC
GGTGCCGCCACCATGCAGCTGAGGAACCCAGAACTACATCTGGGCTGCGCGCTTGCGCTTCGCTTCCTGGCCCTCGT
TTCCTGGGACATCCCTGGGGCTAGAGCACTGGACAATGGATTGGCAAGGACGCCTACCATGGGCTGGCTGCACTGG
GAGCGCTTCATGTGCAACCTTGACTGCCAGGAAGAGCCAGATTCCTGCATCAGTGAGAAGCTCTTCATGGAGATGG
CAGAGCTCATGGTCTCAGAAGGCTGGAAGGATGCAGGTTATGAGTACCTCTGCATTGATGACTGTTGGATGGCTCC
CCAAAGAGATTCAGAAGGCAGACTTCAGGCAGACCCTCAGCGCTTTCCTCATGGGATTCGCCAGCTAGCTAATTATG
TTCACAGCAAAGGACTGAAGCTAGGGATTTATGCAGATGTTGGAAATAAAACCTGCGCAGGCTTCCCTGGGAGTTTT
GGATACTACGACATTGATGCCCAGACCTTTGCTGACTGGGGAGTAGATCTGCTAAAATTTGATGGTTGTTACTGTGA
CAGTTTGGAAAATTTGGCAGATGGTTATAAGCACATGTCCTTGGCCCTGAATAGGACTGGCAGAAGCATTGTGTACT
CCTGTGAGTGGCCTCTTTATATGTGGCCCTTTCAAAAGCCCAATTATACAGAAATCCGACAGTACTGCAATCACTGGC
GAAATTTTGCTGACATTGATGATTCCTGGAAAAGTATAAAGAGTATCTTGGACTGGACATCTTTTAACCAGGAGAGA
ATTGTTGATGTTGCTGGACCAGGGGGTTGGAATGACCCAGATATGTTAGTGATTGGCAACTTTGGCCTCAGCTGGA
ATCAGCAAGTAACTCAGATGGCCCTCTGGGCTATCATGGCTGCTCCTTTATTCATGTCTAATGACCTCCGACACATCA
GCCCTCAAGCCAAAGCTCTCCTTCAGGATAAGGACGTAATTGCCATCAATCAGGACCCCTTGGGCAAGCAAGGGTAC
CAGCTTAGACAGGGAGACAACTTTGAAGTGTGGGAACGACCTCTCTCAGGCTTAGCCTGGGCTGTAGCTATGATAA
ACCGGCAGGAGATTGGTGGACCTCGCTCTTATACCATCGCAGTTGCTTCCCTGGGTAAAGGAGTGGCCTGTAATCCT
GCCTGCTTCATCACACAGCTCCTCCCTGTGAAAAGGAAGCTAGGGTTCTATGAATGGACTTCAAGGTTAAGAAGTCA
CATAAATCCCACAGGCACTGTTTTGCTTCAGCTAGAAAATACAATGCAGATGTCATTAAAAGACTTACTTTAAAGCTT
GCTCGCTTTCTTGCTGTCCAATTTCTATTAAAGGTTCCTTTGTTCCCTAAGTCCAACTACTAAACTGGGGGATATTATG
AAGGGCCTTGAGCATCTGGATTCTGCCTAATAAAAAACATTTATTTTCATTGCTAGCAAAAAAAAAAAAAAAAAAAA
AAAAAAAAAAAAAAAAAAAAAAAAAAAAAAAAAAAAAAGAAAAAAAAAAAAAAAAAAAAAAAAAAAAAAAAA
AAAAAAAAAAAAAAAAAAAAAAAAAACGTCTTCCTGCAGGGATCCCTGGGCCTCATGGGCCTTCCGCTCACTGCCC
GCTTTCCAGTCGGGAAACCTGTCGTGCCAGCTGCATTAACATGGTCATAGCTGTTTCCTTGCGTATTGGGCGCTCTCC
GCTTCCTCGCTCACTGACTCGCTGCGCTCGGTCGTTCGGGTAAAGCCTGGGGTGCCTAATGAGCAAAAGGCCAGCA
AAAGGCCAGGAACCGTAAAAAGGCCGCGTTGCTGGCGTTTTTCCATAGGCTCCGCCCCCCTGACGAGCATCACAAA
AATCGACGCTCAAGTCAGAGGTGGCGAAACCCGACAGGACTATAAAGATACCAGGCGTTTCCCCCTGGAAGCTCCC
TCGTGCGCTCTCCTGTTCCGACCCTGCCGCTTACCGGATACCTGTCCGCCTTTCTCCCTTCGGGAAGCGTGGCGCTTTC
TCATAGCTCACGCTGTAGGTATCTCAGTTCGGTGTAGGTCGTTCGCTCCAAGCTGGGCTGTGTGCACGAACCCCCCG
TTCAGCCCGACCGCTGCGCCTTATCCGGTAACTATCGTCTTGAGTCCAACCCGGTAAGACACGACTTATCGCCACTGG
CAGCAGCCACTGGTAACAGGATTAGCAGAGCGAGGTATGTAGGCGGTGCTACAGAGTTCTTGAAGTGGTGGCCTA
ACTACGGCTACACTAGAAGAACAGTATTTGGTATCTGCGCTCTGCTGAAGCCAGTTACCTTCGGAAAAAGAGTTGGT
AGCTCTTGATCCGGCAAACAAACCACCGCTGGTAGCGGTGGTTTTTTTGTTTGCAAGCAGCAGATTACGCGCAGAAA
AAAAGGATCTCAAGAAGATCCTTTGATCTTTTCTACGGGGTCTGACGCTCAGTGGAACGAAAACTCACGTTAAGGGA
TTTTGGTCATGAGATTATCAAAAAGGATCTTCACCTAGATCCTTTTAAATTAAAAATGAAGTTTTAAATCAATCTAAA
GTATATATGAGTAAACTTGGTCTGACAGTTACCAATGCTTAATCAGTGAGGCACCTATCTCAGCGATCTGTCTATTTC
GTTCATCCATAGTTGCCTGACTCCCCGTCGTGTAGATAACTACGATACGGGAGGGCTTACCATCTGGCCCCAGTGCT
GCAATGATACCGCGAGAACCACGCTCACCGGCTCCAGATTTATCAGCAATAAACCAGCCAGCCGGAAGGGCCGAGC
GCAGAAGTGGTCCTGCAACTTTATCCGCCTCCATCCAGTCTATTAATTGTTGCCGGGAAGCTAGAGTAAGTAGTTCG
CCAGTTAATAGTTTGCGCAACGTTGTTGCCATTGCTACAGGCATCGTGGTGTCACGCTCGTCGTTTGGTATGGCTTCA
TTCAGCTCCGGTTCCCAACGATCAAGGCGAGTTACATGATCCCCCATGTTGTGCAAAAAAGCGGTTAGCTCCTTCGG

TCCTCCGATCGTTGTCAGAAGTAAGTTGGCCGCAGTGTTATCACTCATGGTTATGGCAGCACTGCATAATTCTCTTAC
TGTCATGCCATCCGTAAGATGCTTTTCTGTGACTGGTGAGTACTCAACCAAGTCATTCTGAGAATAGTGTATGCGGC
GACCGAGTTGCTCTTGCCCGGCGTCAATACGGGATAATACCGCGCCACATAGCAGAACTTTAAAAGTGCTCATCATT
GGAAAACGTTCTTCGGGGCGAAAACTCTCAAGGATCTTACCGCTGTTGAGATCCAGTTCGATGTAACCCACTCGTGC
ACCCAACTGATCTTCAGCATCTTTTACTTTCACCAGCGTTTCTGGGTGAGCAAAAACAGGAAGGCAAAATGCCGCAA
AAAAGGGAATAAGGGCGACACGGAAATGTTGAATACTCATACTCTTCCTTTTTCAATATTATTGAAGCATTTATCAGG
GTTATTGTCTCATGAGCGGATACATATTTGAATGTATTTAGAAAAATAAACAAATAGGGGTTCCGCGCACATTTCCCC
GAAAAGTGCCAC

>modGLA

Cap1GGAGAACUCUUCUGGUCCCCACAGACUCAGAGAGAACCCACCGGUGCCGCCACCAUGCAGCUGAGGAACC
CAGAACUACAUCUGGGCUGCGCGCUUGCGCUUCGCUUCCUGGCCCUCGUUUCCUGGGACAUCCCUGGGGCUA
GAGCACUGGACAAUGGAUUGGCAAGGACGCCUACCAUGGGCUGGCUGCACUGGGAGCGCUUCAUGUGCAACC
UUGACUGCCAGGAAGAGCCAGAUUCCUGCAUCAGUGAGAAGCUCUUCAUGGAGAUGGCAGAGCUCAUGGUCU
CAGAAGGCUGGAAGGAUGCAGGUUAUGAGUACCUCUGCAUUGAUGACUGUUGGAUGGCUCCCCAAAGAGAU
UCAGAAGGCAGACUUCAGGCAGACCCUCAGCGCUUUCCUCAUGGGAUUCGCCAGCUAGCUAAUUAUGUUCAC
AGCAAAGGACUGAAGCUAGGGAUUUAUGCAGAUGUUGGAAAUAAAACCUGCGCAGGCUUCCCUGGGAGUUU
UGGAUACUACGACAUUGAUGCCCAGACCUUUGCUGACUGGGGAGUAGAUCUGCUAAAAUUUGAUGGUUGUU
ACUGUGACAGUUUGGAAAAUUUGGCAGAUGGUUAUAAGCACAUGUCCUUGGCCCUGAAUAGGACUGGCAGA
AGCAUUGUGUACUCCUGUGAGUGGCCUCUUUAUAUGUGGCCCUUUCAAAAGCCCAAUUAUACAGAAAUCCGA
CAGUACUGCAAUCACUGGCGAAAUUUUGCUGACAUUGAUGAUUCCUGGAAAAGUAUAAAGAGUAUCUUGGA
CUGGACAUCUUUUAACCAGGAGAGAAUUGUUGAUGUUGCUGGACCAGGGGGUUGGAAUGACCCAGAUAUGU
UAGUGAUUGGCAACUUUGGCCUCAGCUGGAAUCAGCAAGUAACUCAGAUGGCCCUCUGGGCUAUCAUGGCUG
CUCCUUUAUUCAUGUCUAAUGACCUCCGACACAUCAGCCCUCAAGCCAAAGCUCUCCUUCAGGAUAAGGACGU
AAUUGCCAUCAAUCAGGACCCCUUGGGCAAGCAAGGGUACCAGCUUAGACAGGGAGACAACUUUGAAGUGUG
GGAACGACCUCUCUCAGGCUUAGCCUGGGCUGUAGCUAUGAUAAACCGGCAGGAGAUUGGUGGACCUCGCUC
UUAUACCAUCGCAGUUGCUUCCCUGGGUAAAGGAGUGGCCUGUAAUCCUGCCUGCUUCAUCACACAGCUCCU
CCCUGUGAAAAGGAAGCUAGGGUUCUAUGAAUGGACUUCAAGGUUAAGAAGUCACAUAAAUCCCACAGGCAC
UGUUUUGCUUCAGCUAGAAAAUACAAUGCAGAUGUCAUUAAAAGACUUACUUUAAAGCUUGCUCGCUUUCU
UGCUGUCCAAUUUCUAUUAAAGGUUCCUUUGUUCCCUAAGUCCAACUACUAAACUGGGGGAUAUUAUGAAG
GGCCUUGAGCAUCUGGAUUCUGCCUAAUAAAAAACAUUUAUUUUCAUUGCUAGCAAAAAAAAAAAAAAAAAA
AAAAAAAAAAAAAAAAAAAAAAAAAAAAAAAAAAAAAAAAAAGAAAAAAAAAAAAAAAAAAAAAAAAAAAAAAA
AAAAAAAAAAAAAAAAAAAAAAAAAAA

>pMIVT.fLuc.BtgZI

CTAAATTGTAAGCGTTAATATTTTGTTAAAATTCGGATGTGCTGCAAGGCGAGCGGCCGCAGGCCGTCAAGGCCGC
ATGGTACCTGACAGCTGTAATACGACTCACTATAAGGAGAACTCTTCTGGTCCCCACAGACTCAGAGAGAACCCACC
GGTGCCACCATGGAAGACGCCAAAAACATAAAGAAAGGCCCGGCGCCATTCTATCCGCTGGAAGATGGAACCGCT
GGAGAGCAACTGCATAAGGCTATGAAGAGATACGCCCTGGTTCCTGGAACAATTGCTTTTACAGATGCACATATCGA
GGTGGACATCACTTACGCTGAGTACTTCGAAATGTCCGTTCGGTTGGCAGAAGCTATGAAACGATATGGGCTGAAT
ACAAATCACAGAATCGTCGTATGCAGTGAAAACTCTCTTCAATTCTTTATGCCGGTGTTGGGCGCGTTATTTATCGGA
GTTGCAGTTGCGCCCGCGAACGACATTTATAATGAACGTGAATTGCTCAACAGTATGGGCATTTCGCAGCCTACCGT
GGTGTTCGTTTCCAAAAAGGGGTTGCAAAAAATTTTGAACGTGCAAAAAAAGCTCCCAATCATCCAAAAAATTATTA
TCATGGATTCTAAAACGGATTACCAGGGATTTCAGTCGATGTACACGTTCGTCACATCTCATCTACCTCCCGGTTTTA
ATGAATACGATTTTGTGCCAGAGTCCTTCGATAGGGACAAGACAATTGCACTGATCATGAACTCCTCTGGATCTACT
GGTCTGCCTAAAGGTGTCGCTCTGCCTCATAGAACTGCCTGCGTGAGATTCTCGCATGCCAGAGATCCTATTTTTGGC
AATCAAATCATTCCGGATACTGCGATTTTAAGTGTTGTTCCATTCCATCACGGTTTTGGAATGTTTACTACACTCGGAT

ATTTGATATGTGGATTTCGAGTCGTCTTAATGTATAGATTTGAAGAAGAGCTGTTTCTGAGGAGCCTTCAGGATTAC
AAGATTCAAAGTGCGCTGCTGGTGCCAACCCTATTCTCCTTCTTCGCCAAAAGCACTCTGATTGACAAATACGATTTA
TCTAATTTACACGAAATTGCTTCTGGTGGCGCTCCCCTCTCTAAGGAAGTCGGGGAAGCGGTTGCCAAGAGGTTCCA
TCTGCCAGGTATCAGGCAAGGATATGGGCTCACTGAGACTACATCAGCTATTCTGATTACACCCGAGGGGGATGAT
AAACCGGGCGCGGTCGGTAAAGTTGTTCCATTTTTTGAAGCGAAGGTTGTGGATCTGGATACCGGGAAAACGCTGG
GCGTTAATCAAAGAGGCGAACTGTGTGTGAGAGGTCCTATGATTATGTCCGGTTATGTAAACAATCCGGAAGCGAC
CAACGCCTTGATTGACAAGGATGGATGGCTACATTCTGGAGACATAGCTTACTGGGACGAAGACGAACACTTCTTCA
TCGTTGACCGCCTGAAGTCTCTGATTAAGTACAAAGGCTATCAGGTGGCTCCCGCTGAATTGGAATCCATCTTGCTCC
AACACCCCAACATCTTCGACGCAGGTGTCGCAGGTCTTCCCGACGATGACGCCGGTGAACTTCCCGCCGCCGTTGTT
GTTTTGGAGCACGGAAAGACGATGACGGAAAAAGAGATCGTGGATTACGTCGCCAGTCAAGTAACAACCGCGAAA
AAGTTGCGCGGAGGAGTTGTGTTTGTGGACGAAGTACCGAAAGGTCTTACCGGAAAACTCGACGCAAGAAAAATC
AGAGAGATCCTCATAAAGGCCAAGAAGGGCGGAAAGATCGCCGTGTAAAAGCTTGCTCGCTTTCTTGCTGTCCAATT
TCTATTAAAGGTTCCTTTGTTCCCTAAGTCCAACTACTAAACTGGGGGATATTATGAAGGGCCTTGAGCATCTGGATT
CTGCCTAATAAAAAACATTTATTTTCATTGCTAGCAAAAAAAAAAAAAAAAAAAAAAAAAAAAAAAAAAAAAAAAAAAA
AAAAAAAAAAAAAAAAAAAGAAAAAAAAAAAAAAAAAAAAAAAAAAAAAAAAAAAAAAAAAAAAAAAAAAAAAAAAA
AAAAAAAGAAGAGCACGCATCGCGGATCCCTGGGCCTCATGGGCCTTCCGCTCACTGCCCGCTTTCCAGTCGGGAA
ACCTGTCGTGCCAGCTGCATTAACATGGTCATAGCTGTTTCCTTGCGTATTGGGCGCTCTCCGCTTCCTCGCTCACTG
ACTCGCTGCGCTCGGTCGTTCGGGTAAAGCCTGGGGTGCCTAATGAGCAAAAGGCCAGCAAAAGGCCAGGAACCG
TAAAAAGGCCGCGTTGCTGGCGTTTTTCCATAGGCTCCGCCCCCCTGACGAGCATCACAAAAATCGACGCTCAAGTC
AGAGGTGGCGAAACCCGACAGGACTATAAAGATACCAGGCGTTTCCCCCTGGAAGCTCCCTCGTGCGCTCTCCTGTT
CCGACCCTGCCGCTTACCGGATACCTGTCCGCCTTTCTCCCTTCGGGAAGCGTGGCGCTTTCTCATAGCTCACGCTGT
AGGTATCTCAGTTCGGTGTAGGTCGTTCGCTCCAAGCTGGGCTGTGTGCACGAACCCCCCGTTCAGCCCGACCGCTG
CGCCTTATCCGGTAACTATCGTCTTGAGTCCAACCCGGTAAGACACGACTTATCGCCACTGGCAGCAGCCACTGGTA
ACAGGATTAGCAGAGCGAGGTATGTAGGCGGTGCTACAGAGTTCTTGAAGTGGTGGCCTAACTACGGCTACACTAG
AAGAACAGTATTTGGTATCTGCGCTCTGCTGAAGCCAGTTACCTTCGGAAAAAGAGTTGGTAGCTCTTGATCCGGCA
AACAAACCACCGCTGGTAGCGGTGGTTTTTTTGTTTGCAAGCAGCAGATTACGCGCAGAAAAAAAGGATCTCAAGA
AGATCCTTTGATCTTTTCTACGGGGTCTGACGCTCAGTGGAACGAAAACTCACGTTAAGGGATTTTGGTCATGAGAT
TATCAAAAAGGATCTTCACCTAGATCCTTTTAAATTAAAAATGAAGTTTTAAATCAATCTAAAGTATATATGAGTAAA
CTTGGTCTGACAGTTACCAATGCTTAATCAGTGAGGCACCTATCTCAGCGATCTGTCTATTTCGTTCATCCATAGTTGC
CTGACTCCCCGTCGTGTAGATAACTACGATACGGGAGGGCTTACCATCTGGCCCCAGTGCTGCAATGATACCGCGAG
AACCACGCTCACCGGCTCCAGATTTATCAGCAATAAACCAGCCAGCCGGAAGGGCCGAGCGCAGAAGTGGTCCTGC
AACTTTATCCGCCTCCATCCAGTCTATTAATTGTTGCCGGGAAGCTAGAGTAAGTAGTTCGCCAGTTAATAGTTTGCG
CAACGTTGTTGCCATTGCTACAGGCATCGTGGTGTCACGCTCGTCGTTTGGTATGGCTTCATTCAGCTCCGGTTCCCA
ACGATCAAGGCGAGTTACATGATCCCCCATGTTGTGCAAAAAAGCGGTTAGCTCCTTCGGTCCTCCGATCGTTGTCA
GAAGTAAGTTGGCCGCAGTGTTATCACTCATGGTTATGGCAGCACTGCATAATTCTCTTACTGTCATGCCATCCGTAA
GATGCTTTTCTGTGACTGGTGAGTACTCAACCAAGTCATTCTGAGAATAGTGTATGCGGCGACCGAGTTGCTCTTGC
CCGGCGTCAATACGGGATAATACCGCGCCACATAGCAGAACTTTAAAAGTGCTCATCATTGGAAAACGTTCTTCGGG
GCGAAAACTCTCAAGGATCTTACCGCTGTTGAGATCCAGTTCGATGTAACCCACTCGTGCACCCAACTGATCTTCAGC
ATCTTTTACTTTCACCAGCGTTTCTGGGTGAGCAAAAACAGGAAGGCAAAATGCCGCAAAAAAGGGAATAAGGGCG
ACACGGAAATGTTGAATACTCATACTCTTCCTTTTTCAATATTATTGAAGCATTTATCAGGGTTATTGTCTCATGAGCG
GATACATATTTGAATGTATTTAGAAAAATAAACAAATAGGGGTTCCGCGCACATTTCCCCGAAAAGTGCCAC

>modfLuc

Cap1GGAGAACUCUUCUGGUCCCCACAGACUCAGAGAGAACCCACCGGUGCCACCAUGGAAGACGCCAAAAACA
UAAAGAAAGGCCCGGCGCCAUUCUAUCCGCUGGAAGAUGGAACCGCUGGAGAGCAACUGCAUAAGGCUAUGA
AGAGAUACGCCCUGGUUCCUGGAACAAUUGCUUUUACAGAUGCACAUAUCGAGGUGGACAUCACUUACGCUG
AGUACUUCGAAAUGUCCGUUCGGUUGGCAGAAGCUAUGAAACGAUAUGGGCUGAAUACAAAUCACAGAAUCG
UCGUAUGCAGUGAAAACUCUCUUCAAUUCUUUAUGCCGGUGUUGGGCGCGUUAUUUAUCGGAGUUGCAGUU

GCGCCCGCGAACGACAUUUAUAAUGAACGUGAAUUGCUCAACAGUAUGGGCAUUUCGCAGCCUACCGUGGUG
UUCGUUUCCAAAAAGGGGUUGCAAAAAAUUUUGAACGUGCAAAAAAAGCUCCCAAUCAUCCAAAAAAUUAUU
AUCAUGGAUUCUAAAACGGAUUACCAGGGAUUUCAGUCGAUGUACACGUUCGUCACAUCUCAUCUACCUCCC
GGUUUUAAUGAAUACGAUUUUGUGCCAGAGUCCUUCGAUAGGGACAAGACAAUUGCACUGAUCAUGAACUCC
UCUGGAUCUACUGGUCUGCCUAAAGGUGUCGCUCUGCCUCAUAGAACUGCCUGCGUGAGAUUCUCGCAUGCC
AGAGAUCCUAUUUUUGGCAAUCAAAUCAUUCCGGAUACUGCGAUUUUAAGUGUUGUUCCAUUCCAUCACGG
UUUUGGAAUGUUUACUACACUCGGAUAUUUGAUAUGUGGAUUUCGAGUCGUCUUAAUGUAUAGAUUUGAA
GAAGAGCUGUUUCUGAGGAGCCUUCAGGAUUACAAGAUUCAAAGUGCGCUGCUGGUGCCAACCCUAUUCUCC
UUCUUCGCCAAAAGCACUCUGAUUGACAAAUACGAUUUAUCUAAUUUACACGAAAUUGCUUCUGGUGGCGCU
CCCCUCUCUAAGGAAGUCGGGGAAGCGGUUGCCAAGAGGUUCCAUCUGCCAGGUAUCAGGCAAGGAUAUGGG
CUCACUGAGACUACAUCAGCUAUUCUGAUUACACCCGAGGGGGAUGAUAAACCGGGCGCGGUCGGUAAAGUU
GUUCCAUUUUUUGAAGCGAAGGUUGUGGAUCUGGAUACCGGGAAAACGCUGGGCGUUAAUCAAAGAGGCGA
ACUGUGUGUGAGAGGUCCUAUGAUUAUGUCCGGUUAUGUAAACAAUCCGGAAGCGACCAACGCCUUGAUUG
ACAAGGAUGGAUGGCUACAUUCUGGAGACAUAGCUUACUGGGACGAAGACGAACACUUCUUCAUCGUUGACC
GCCUGAAGUCUCUGAUUAAGUACAAAGGCUAUCAGGUGGCUCCCGCUGAAUUGGAAUCCAUCUUGCUCCAAC
ACCCCAACAUCUUCGACGCAGGUGUCGCAGGUCUUCCCGACGAUGACGCCGGUGAACUUCCCGCCGCCGUUGU
UGUUUUGGAGCACGGAAAGACGAUGACGGAAAAAGAGAUCGUGGAUUACGUCGCCAGUCAAGUAACAACCGC
GAAAAAGUUGCGCGGAGGAGUUGUGUUUGUGGACGAAGUACCGAAAGGUCUUACCGGAAAACUCGACGCAA
GAAAAAUCAGAGAGAUCCUCAUAAAGGCCAAGAAGGGCGGAAAGAUCGCCGUGUAAAAGCUUGCUCGCUUUC
UUGCUGUCCAAUUUCUAUUAAAGGUUCCUUUGUUCCCUAAGUCCAACUACUAAACUGGGGGAUAUUAUGAA
GGGCCUUGAGCAUCUGGAUUCUGCCUAAUAAAAAACAUUUAUUUUCAUUGCUAGCAAAAAAAAAAAAAAAAA
AAAAAAAAAAAAAAAAAAAAAAAAAAAAAAAAAAAAAAAAAAGAAAAAAAAAAAAAAAAAAAAAAAAAAAAA
AAAAAAAAAAAAAAAAAAAAAAAAAAAAAA

>pMIVT.eGFP.BspQI

CTAAATTGTAAGCGTTAATATTTTGTTAAAATTCGGATGTGCTGCAAGGCGAGCGGCCGCAGGCCGTCAAGGCCGC
ATGGTACCTGACAGCTGTAATACGACTCACTATAAGGAGAACTCTTCTGGTCCCCACAGACTCAGAGAGAACCCACC
GGTGCCGCCACCATGGTGAGCAAGGGCGAGGAGCTGTTCACCGGGGTGGTGCCCATCCTGGTCGAGCTGGACGGC
GACGTAAACGGCCACAAGTTCAGCGTGTCCGGCGAGGGCGAGGGCGATGCCACCTACGGCAAGCTGACCCTGAAG
TTCATCTGCACCACCGGCAAGCTGCCCGTGCCCTGGCCCACCCTCGTGACCACCCTGACCTACGGCGTGCAGTGCTTC
AGCCGCTACCCCGACCACATGAAGCAGCACGACTTCTTCAAGTCCGCCATGCCCGAAGGCTACGTCCAGGAGCGCA
CCATCTTCTTCAAGGACGACGGCAACTACAAGACCCGCGCCGAGGTGAAGTTCGAGGGCGACACCCTGGTGAACCG
CATCGAGCTGAAGGGCATCGACTTCAAGGAGGACGGCAACATCCTGGGGCACAAGCTGGAGTACAACTACAACAG
CCACAACGTCTATATCATGGCCGACAAGCAGAAGAACGGCATCAAGGTGAACTTCAAGATCCGCCACAACATCGAG
GACGGCAGCGTGCAGCTCGCCGACCACTACCAGCAGAACACCCCCATCGGCGACGGCCCCGTGCTGCTGCCCGACA
ACCACTACCTGAGCACCCAGTCCGCCCTGAGCAAAGACCCCAACGAGAAGCGCGATCACATGGTCCTGCTGGAGTT
CGTGACCGCCGCCGGGATCACTCTCGGCATGGACGAGCTGTACAAGTAAGCTTGCTCGCTTTCTTGCTGTCCAATTTC
TATTAAAGGTTCCTTTGTTCCCTAAGTCCAACTACTAAACTGGGGGATATTATGAAGGGCCTTGAGCATCTGGATTCT
GCCTAATAAAAAACATTTATTTTCATTGCTAGCAAAAAAAAAAAAAAAAAAAAAAAAAAAAAAAAAAAAA
AAAAAAAAAAAAAAAAAGAAAAAAAAAAAAAAAAAAAAAAAAAAAAAAAAAAAAAAAAAAAAAAAAAAAA
AAAACGAAGAGCCTGCAGTGAGGATCCCTGGGCCTCATGGGCCTTCCGCTCACTGCCCGCTTTCCAGTCGGGAAACC
TGTCGTGCCAGCTGCATTAACATGGTCATAGCTGTTTCCTTGCGTATTGGGCGCTCTCCGCTTCCTCGCTCACTGACTC
GCTGCGCTCGGTCGTTCGGGTAAAGCCTGGGGTGCCTAATGAGCAAAAGGCCAGCAAAAGGCCAGGAACCGTAAA
AAGGCCGCGTTGCTGGCGTTTTTCCATAGGCTCCGCCCCCCTGACGAGCATCACAAAAATCGACGCTCAAGTCAGAG
GTGGCGAAACCCGACAGGACTATAAAGATACCAGGCGTTTCCCCCTGGAAGCTCCCTCGTGCGCTCTCCTGTTCCGA
CCCTGCCGCTTACCGGATACCTGTCCGCCTTTCTCCCTTCGGGAAGCGTGGCGCTTTCTCATAGCTCACGCTGTAGGT
ATCTCAGTTCGGTGTAGGTCGTTCGCTCCAAGCTGGGCTGTGTGCACGAACCCCCCGTTCAGCCCGACCGCTGCGCC
TTATCCGGTAACTATCGTCTTGAGTCCAACCCGGTAAGACACGACTTATCGCCACTGGCAGCAGCCACTGGTAACAG

GATTAGCAGAGCGAGGTATGTAGGCGGTGCTACAGAGTTCTTGAAGTGGTGGCCTAACTACGGCTACACTAGAAGA
ACAGTATTTGGTATCTGCGCTCTGCTGAAGCCAGTTACCTTCGGAAAAAGAGTTGGTAGCTCTTGATCCGGCAAACA
AACCACCGCTGGTAGCGGTGGTTTTTTTGTTTGCAAGCAGCAGATTACGCGCAGAAAAAAAGGATCTCAAGAAGAT
CCTTTGATCTTTTCTACGGGGTCTGACGCTCAGTGGAACGAAAACTCACGTTAAGGGATTTTGGTCATGAGATTATCA
AAAAGGATCTTCACCTAGATCCTTTTAAATTAAAAATGAAGTTTTAAATCAATCTAAAGTATATATGAGTAAACTTGG
TCTGACAGTTACCAATGCTTAATCAGTGAGGCACCTATCTCAGCGATCTGTCTATTTCGTTCATCCATAGTTGCCTGAC
TCCCCGTCGTGTAGATAACTACGATACGGGAGGGCTTACCATCTGGCCCCAGTGCTGCAATGATACCGCGAGAACCA
CGCTCACCGGCTCCAGATTTATCAGCAATAAACCAGCCAGCCGGAAGGGCCGAGCGCAGAAGTGGTCCTGCAACTT
TATCCGCCTCCATCCAGTCTATTAATTGTTGCCGGGAAGCTAGAGTAAGTAGTTCGCCAGTTAATAGTTTGCGCAACG
TTGTTGCCATTGCTACAGGCATCGTGGTGTCACGCTCGTCGTTTGGTATGGCTTCATTCAGCTCCGGTTCCCAACGAT
CAAGGCGAGTTACATGATCCCCCATGTTGTGCAAAAAAGCGGTTAGCTCCTTCGGTCCTCCGATCGTTGTCAGAAGT
AAGTTGGCCGCAGTGTTATCACTCATGGTTATGGCAGCACTGCATAATTCTCTTACTGTCATGCCATCCGTAAGATGC
TTTTCTGTGACTGGTGAGTACTCAACCAAGTCATTCTGAGAATAGTGTATGCGGCGACCGAGTTGCTCTTGCCCGGC
GTCAATACGGGATAATACCGCGCCACATAGCAGAACTTTAAAAGTGCTCATCATTGGAAAACGTTCTTCGGGGCGAA
AACTCTCAAGGATCTTACCGCTGTTGAGATCCAGTTCGATGTAACCCACTCGTGCACCCAACTGATCTTCAGCATCTTT
TACTTTCACCAGCGTTTCTGGGTGAGCAAAAACAGGAAGGCAAAATGCCGCAAAAAAGGGAATAAGGGCGACACG
GAAATGTTGAATACTCATACTCTTCCTTTTTCAATATTATTGAAGCATTTATCAGGGTTATTGTCTCATGAGCGGATAC
ATATTTGAATGTATTTAGAAAAATAAACAAATAGGGGTTCCGCGCACATTTCCCCGAAAAGTGCCAC

>modEGFP

Cap1GGAGAACUCUUCUGGUCCCCACAGACUCAGAGAGAACCCACCGGUGCCGCCACCAUGGUGAGCAAGGGCG
AGGAGCUGUUCACCGGGGUGGUGCCCAUCCUGGUCGAGCUGGACGGCGACGUAAACGGCCACAAGUUCAGCG
UGUCCGGCGAGGGCGAGGGCGAUGCCACCUACGGCAAGCUGACCCUGAAGUUCAUCUGCACCACCGGCAAGCU
GCCCGUGCCCUGGCCCACCCUCGUGACCACCCUGACCUACGGCGUGCAGUGCUUCAGCCGCUACCCCGACCACA
UGAAGCAGCACGACUUCUUCAAGUCCGCCAUGCCCGAAGGCUACGUCCAGGAGCGCACCAUCUUCUUCAAGGA
CGACGGCAACUACAAGACCCGCGCCGAGGUGAAGUUCGAGGGCGACACCCUGGUGAACCGCAUCGAGCUGAAG
GGCAUCGACUUCAAGGAGGACGGCAACAUCCUGGGGCACAAGCUGGAGUACAACUACAACAGCCACAACGUCU
AUAUCAUGGCCGACAAGCAGAAGAACGGCAUCAAGGUGAACUUCAAGAUCCGCCACAACAUCGAGGACGGCAG
CGUGCAGCUCGCCGACCACUACCAGCAGAACACCCCCAUCGGCGACGGCCCCGUGCUGCUGCCCGACAACCACU
ACCUGAGCACCCAGUCCGCCCUGAGCAAAGACCCCAACGAGAAGCGCGAUCACAUGGUCCUGCUGGAGUUCGU
GACCGCCGCCGGGAUCACUCUCGGCAUGGACGAGCUGUACAAGUAAGCUUGCUCGCUUUCUUGCUGUCCAAU
UUCUAUUAAAGGUUCCUUUGUUCCCUAAGUCCAACUACUAAACUGGGGGAUAUUAUGAAGGGCCUUGAGCA
UCUGGAUUCUGCCUAAUAAAAAACAUUUAUUUUCAUUGCUAGCAAAAAAAAAAAAAAAAAAAAAAAAAAAA
AAAAAAAAAAAAAAAAAAAAAAAAAAAAAAGAAAAAAAAAAAAAAAAAAAAAAAAAAAAAAAAAAAAAAAAAA
AAAAAAAAAAAAAAAAAA
